# Innovative Approaches for Recovery of Phytoconstituents from Medicinal/Aromatic Plants and Biotechnological Production

**DOI:** 10.3390/molecules25020309

**Published:** 2020-01-12

**Authors:** Radu Claudiu Fierascu, Irina Fierascu, Alina Ortan, Milen I. Georgiev, Elwira Sieniawska

**Affiliations:** 1 University of Agronomic Science and Veterinary Medicine, 59 Marasti Blvd., 011464 Bucharest, Romania; radu_claudiu_fierascu@yahoo.com (R.C.F.); alina_ortan@hotmail.com (A.O.); milengeorgiev@gbg.bg (M.I.G.); 2 National Institute for Research & Development in Chemistry and Petrochemistry, ICECHIM Bucharest, 202 Spl. Independentei, 060021 Bucharest, Romania; 3 Group of Plant Cell Biotechnology and Metabolomics, Institute of Microbiology, Bulgarian Academy of Sciences, 139 Ruski Blvd., 4000 Plovdiv, Bulgaria; 4 Department of Pharmacognosy with Medicinal Plant Unit, Medical University of Lublin, 1 Chodzki, 20-093 Lublin, Poland; esieniawska@pharmacognosy.org

**Keywords:** medicinal and aromatic plants, bioactive compounds, plant biotechnology, solid-liquid extraction, liquid-liquid separation

## Abstract

Continuously growing demand for plant derived therapeutic molecules obtained in a sustainable and eco-friendly manner favors biotechnological production and development of innovative extraction techniques to obtain phytoconstituents. What is more, improving and optimization of alternative techniques for the isolation of high value natural compounds are issues having both social and economic importance. In this critical review, the aspects regarding plant biotechnology and green downstream processing, leading to the production and extraction of increased levels of fine chemicals from both plant cell, tissue, and organ culture or fresh plant materials and the remaining by-products, are discussed.

## 1. Introduction

Plants have been used since the beginning of the human civilization for healing purposes. Medicinal plants are related to different types of traditional medicine, like Traditional Chinese Medicine, Indian Ayurveda, or Japanese Kampo, but in the rest of the world, they are in most of the cases complementary to drug use. The last few decades have witnessed a substantial increase in herbal products markets throughout the world, with many end-uses such as flavors, colorants, essential oils, sweeteners, antioxidants or nutraceuticals [1]. Over 8000 phenolic compounds derived from medicinal plants are being used currently in the phytotherapy [2] in the form of herbal teas, traditional and new medicines, industrial/pharmaceutical auxiliary products, functional foods, and galenic products.

Medicinal and aromatic plants (MAPs) represent an inexhaustible source of life saving drugs for the majority of the world’s population. The issues created by the increase of the human population, together with a reduction in renewable resources, is reflected in the increase of the global demand for medicinal plants [3]. In this light, the continuously growing demand for therapeutic molecules, produced by “green processes”, and decreasing the quantity of wastes are premises for the development of alternative approaches for sustainable production of phytopharmaceuticals from plants and plants waste. Industrial biotechnology appears to be a promising tool for this purpose. Additionally, the development and optimization of alternative techniques for the isolation of high value phytoconstituents are issues having both social and economic importance. In this context, current research is focused on two approaches, which can be used to obtain phytochemicals. The first alternative approach is represented by the biotechnological techniques, leading to the production of plants with increased levels of fine chemicals, new compounds with potential biological activity, colorants or fragrances, etc. [1]. The second approach is to develop greener extraction techniques to obtain bioactive compounds from fresh plant material or the remaining wastes. The exponentially increasing demand for bioactive compounds led to the development of complex extraction techniques and methods. Conventional extraction techniques, with simple instrumentation, were used at a large scale, but, as a consequence of the long processing time, high solvent and energy consumption required, were replaced with more complex and modern ones. These innovative techniques, based on microwave power, pressurized liquid extraction, or ultrasound are emerging technologies, which can significantly overcome the disadvantages of classical methods (such as the need for large quantities of raw material with decreased yields of active compound recovery). Furthermore, non-conventional techniques such as electrotechnologies (high voltage electric discharge, pulsed electric field, ohmic assisted extraction) are promising tools for the isolation of bioactive compounds from plant material [4].

The extraction techniques applied to various vegetal materials became the focus of an increasing number of studies from all over the world in the last few decades, as proven by the survey performed using several databases (Scopus, PubMed, Medline, ScienceDirect). In this critical review, aspects regarding plant biotechnology, permitting the production of plants with increased levels of fine chemicals, and greener downstream processing methods, in order to obtain bioactive compounds from both fresh plant materials and the remaining by-products, are discussed. These techniques are described, presenting both advantages and disadvantages, also offering significant examples.

The definitions of the essential terms and techniques used in the following paragraphs are presented in Table 1.

## 2. Challenges in the Process “from Plants to Pure Molecules”

The path “from plants to pure molecules” is a rather complex issue: it is influenced by a wide range of factors, from ecological elements of the environment to threats of the modern society, urbanization, and industry development. Furthermore, the amount of active principles in the plant is influenced by ecological factors, species, zoning, culture technology, the biological value of the cultivar, and processing methods. The unambiguous identification is often difficult due to changes in plant taxonomy [5]. Some species of medicinal plants are threatened by over-harvesting in several parts of the world. This is the case, for instance, of Golden Root (*Rhodiola rosea* L.), commonly used in traditional medicine as a cure for several diseases, including anxiety or depression [6,7]. *Rhodiola rosea* L. is included in the Law of Biodiversity (e.g., in Bulgaria), and its harvesting is now forbidden.

The most significant threat to wild medicinal herbs is habitat loss through residential and commercial development (including urbanization, industrialization, and tourism development). The impact of agriculture was identified as another significant threat [8]. Even if these threats are on a rising slope, there are countries with a very long tradition in medicinal and aromatic plants’ cultivation currently developing this sector on large areas. At the European level, several countries are in the foreground of MAP cultivation such as Bulgaria, France, Poland, Hungary, or Romania with species cultivated on over 25,000 ha each: to 2030, the total available land for MAP cultivation is expected to reach 26.2 Mha. Spain is considered to possess the largest available land in 2020 (3616 ha), while Poland will be the leading cultivator in 2030 (4079 ha). Spain, Germany, Poland, France, and Romania are the top five MAP cultivating countries. More than 80% of the total land available for non-food crops is provided by these five countries together with Italy, Bulgaria, and Hungary. These eight European countries will continuously increase this contribution to 81.7% and 84.5%, in 2020 and 2030, respectively [9].

Overcollection of species possesses a significant impact on some commercially valuable wild species and their habitats. A classic example is represented by the “Taxol supply crisis”: when the compound was proven to possess clinical efficacy in cancer treatment, the demand for it greatly increased [10].

Wild and cultivated medicinal and aromatic plants pass through a complex process up to the production phase, which involves several stages such as identification and preliminary screening, primary processing and advanced processing, followed by secondary metabolites’ isolation, characterization, and finally massive production. This process must respect EU regulations in the case of EU countries or other particular regulations, specific to the country where plants are processed. At the EU level, the European Pharmacopoeia provides specific instructions on herbal drug preparations, as well as on aspects such as methods, tests, identification, assays, and possible contaminants [11].

Specific labeling of the traditional herbal medicinal products is defined in the European and national legislation. The requirements for applications for marketing authorization or registration of herbal medicinal products in the EU are very demanding, and according to EU legislation, they must contain information regarding: quality control, good manufacturing practice (GMP), good agricultural and collection practice (GACP), new tests, safety, traditional use, efficacy, consumer information, labeling and advertising, and pharmacovigilance [11]. As stated by Carvalho et al. [12], there are 10,000 licensed herbal medicinal products (HMP) in Germany, 25% of which are combined formulations. In the United Kingdom, there are 3000 licensed HMP, 10% of which are traditional products [12].

As with any drug, clinical trials for safety, efficacy, and/or effectiveness are the last proof before therapeutic use of herbal products. The outcome of the treatment with herbal medicines is mainly dependent on the patient’s participation.

## 3. An Overview of the Biotechnological Aspects for Obtaining Phytochemicals from MAP

More than a century has passed since the very first pioneering attempt of Gottlieb Haberlandt (1902) to grow isolated plant cells in vitro. Currently, in vitro plant technologies, through which plant cells, tissues, and organs (the so-called “green cell factories” concept) are grown artificially in shaken flasks and bioreactors, are considered as cost effective and eco-friendly alternatives to classical approaches (i.e., wild harvest) for the mass production of plant derived molecules, due to their several advantages [13]. First, the bioprocess is fully independent of any seasonal and geographical conditions. Second, genetic modifications (including gene/transcriptional factors overexpression, RNA interference, and application of recently emerging clustered regularly interspaced short palindromic repeats (CRISPR)-Cas for genome editing in a contained system) can readily be applied without the regulatory barriers associated with the field grown plants. Third, a plant cell, tissue, and organ culture (PCTOC) system can be up-scaled in bioreactors with eventually controllable production titers [14]. Furthermore, PCTOC appears as the only economically feasible way of producing some high value metabolites (in general, secondary metabolites represent <1% of the plant cell dry weight) from rare and threatened plants and, in particular, from MAPs. The progress in this field has resulted in the mass production of several pharmaceutically relevant metabolites, most notably paclitaxel (Taxol™), shikonin, galantamine, camptothecin, and artemisinin, besides ginseng biomass and *Echinacea* polysaccharides [15,16,17]. Dozens of commercial processes were developed accordingly and several others and on the pipeline at present [15,18].

A notable example, worth being mentioned, is the paclitaxel bioproduction process. Initially, the paclitaxel demand (ca. 25 kg of drug/year) was met by harvesting the bark of *Taxus brevifolia*. However, because of the low paclitaxel content in the bark (<0.02% of the dry weight), it is estimated that 340 tons of *Taxus* bark (or 38.000 trees) are required to obtain the desired amount of the compound [15]. Currently, in Germany, Phyton Biotech operates the world’s biggest certified GMP PCTOC facility with a cascade of five stirred tank bioreactors (75, 750, 7500, 15,000, and 75,000 L). It is estimated that the total production capacity of the taxanes train runs is up to 880,000 L/year. Phyton Biotech appears as a global provider of chemotherapeutic agents including paclitaxel, docetaxel APIs (active pharmaceutical ingredients), and taxane intermediates [19]. The *Taxus* in vitro based paclitaxel production was also commercialized by Samyang Genex Corporation (South Korea) again at the m^3^ scale [20]. In addition to the relatively well established bioproduction of plant derived molecules, in recent years, several biotech companies have been turning PCTOC into factories to produce food and cosmetics additives. It was recently estimated that over 50 products, based on extracts from PCTOC, are currently used in the cosmetics industry [18].

Another notable example is the utilization of the biosynthetic potential of the adventitious root culture of ginseng. *Panax ginseng* C. A. Meyer (ginseng) is a well known medicinal plant, widely used in the eastern countries for multiple purposes. The use of PCTOC processes has been sought as a potential alternative for the efficient production of ginseng and hence mass supply of raw material for various purposes [21]. PCTOC of ginseng have been developed by CBN Biotecho Company (South Korea) for large scale production of biomass and commercialization. The operation facility, consisting of four 10 m^3^ bioreactors, allows production of ca. 35 tons of ginseng adventitious roots per year, which is a remarkable example of the application of PCTOC to meet the needs of the pharmaceutical, food, and cosmetics industries [21].

A cost effective bioproduction strategy should provide optimal conditions for PCTOC growth, while keeping maximal volumetric productivity of the target compound(s), resulting in eventually minimum operational time. A major drawback of the batch processes is that a significant time is taken by filling up (and emptying) the bioreactor system, cleaning of the vessel, and its further sterilization [17]. Towards enhancement of the cost effectiveness of PCTOC based processes, various operational strategies have been developed so far, such as a multi-stage batch, fed-batch, (semi)continuous, and perfusion regime [16].

Towards a wider industrialization of PCTOC based bioprocesses, several challenges are still to be addressed, mostly related to the relatively high sensitivity of plant cells to different stress factors/conditions and hence better understanding of the cellular/molecular responses to the bioreactor environment. The influence of the cultivation conditions in bioreactors was studied mainly for single compound bioproduction (e.g., rosmarinic acid, for instance), while the impact on the whole metabolic network has been scarcely studied.

Among other valuable tools in systems biology (such as genomics, proteomics, or phenotyping), metabolomics (either in targeted or non-targeted mode) is generally defined as the comprehensive qualitative and quantitative measurement of all metabolites in an organism, at given conditions. The metabolomics platform (e.g., mass spectrometry and nuclear magnetic resonance based) is a powerful tool to gain macroscopic insights into the metabolome of a PCTOC and is of great interest for systems biology studies in all kinds of biological processes [22]. Currently, in the postgenomic era, systems biology opens novel avenues to explore the total biochemical machinery of living systems fully (and in particular PCTOC). Combined metabolite profiling (i.e., targeted metabolomics) and network analysis of PCTOC based processes have revealed that the bioreactor cultivation mode strongly influences the biomass accumulation and production titer of a wide range of high value plant secondary metabolites. Even though further systems biology studies are needed, it is yet clear that the chemical fingerprints left by metabolic processes of plant cells are likely to serve as a powerful tool to understand the molecular responses to the bioreactor environment. This could be further used towards a better management and optimization of PCTOC based bioprocesses [17]. All examples of the application of PCTOC for mass production of biomass and therapeutic molecules, summarized above, clearly outlined their immense potential of upstream plant biotechnologies for future wider industrialization.

## 4. Recovery of Phytochemicals from Biotechnological Production

Biotechnological production of valuable compounds requires the end-product removal from the reaction broth. The downstream processing is usually more expensive than the reaction, so the reduction in the number of necessary steps to obtain pure end-product is advantageous and should be preceded by the choice and design of extraction and purification [23]. The extracellular reactions are more desirable and more often performed due to lower cost and lower number of downstream stages. The soluble or suspended products have to be separated from the cells and then recovered, usually by two phase liquid-liquid extraction (LLE), supercritical fluid extraction, distillation of volatiles (hydro-distillation), chromatography (counter current chromatography, centrifugal partition chromatography), and to a lesser extent, by reactive extraction and complexation [24,25,26]. The protocols resulting in the production of secondary metabolites inside the tissue/microorganism cells involve the biomass extraction. This process follows the principles of solid-liquid extraction (SLE), resulting in the release of the target product with or without disruption of the cells. The most efficient techniques applied for this purpose are microwave assisted extraction (MAE), cavitation accelerated extraction (CAE), and ultrasound assisted extraction (UAE), sometimes preceded by the homogenization process. Besides the financial issues, this additional step complicates downstream processing because it generates “impurities” of the membrane, cytoplasmic, and periplasmatic components, which are not present in extracellular reactions [27]. To obtain the end product with high purity, the LLE or chromatography is also required at this stage.

### 4.1. Non-Disruptive Processing

#### 4.1.1. Liquid-Liquid Extraction

**Figure 1:**
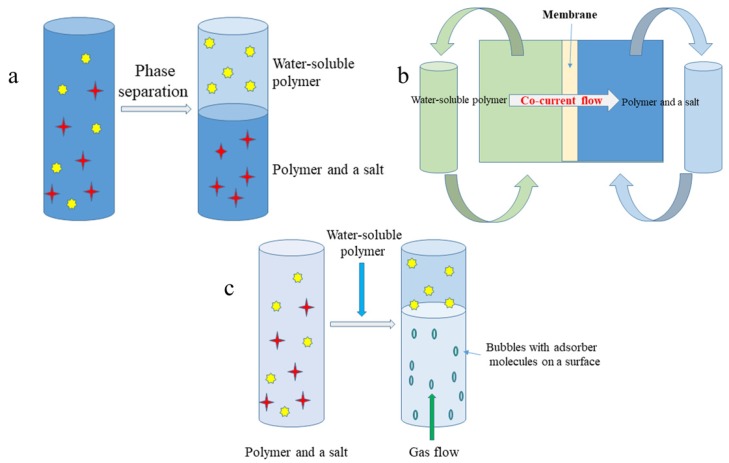
Schematic representation of: (**a**) aqueous two phase system (ATPS) one step liquid-liquid extraction; (**b**) membrane supported liquid-liquid extraction (Memex); (**c**) aqueous two phase flotation (ATPF) system.

The aqueous two phase system (ATPS) one step liquid-liquid extraction (Figure 1a) is the most classical approach to the recovery of biomolecules. Due to its suitability for separation and purification of active secondary metabolites, proteins, enzymes, or cell organelles, it found application in biotechnology [28]. The ATPS is formed by two water soluble polymers (e.g., polyethylene glycol/dextran) or a polymer and a salt (e.g., polyethylene glycol/phosphate) with the presence of more than 80% of water in both phases. ATPS provides fast separation of phases, rapid mass transfer caused by low interfacial tension, and selective separation of compounds without or with little denaturation [29]. ATPS liquid-liquid extraction can be operated as one step batch extraction or as a chain of extraction units, in which a crude biomolecule is partitioned between phases. The equilibrium relationship of the system determines the partition coefficient (K) describing the affinity of the compound to the phases:

(1) $$K=\frac{C_{AT}}{C_{AB}}$$

where *C_AT_* is the equilibrium concentration of component *A* in the top phase and *C_AB_* is the equilibrium concentration of *A* in the lower phase. The volume ratio of the phases, *R* (volume top/volume bottom), and the partition coefficient of the target biomolecule give a theoretical yield in the top or bottom phase (*Y_T_, Y_B_*) [30]:

(2) $$Y_{T}=\frac{V_{T}C_{AT}}{V_{T}C_{AT}+V_{B}C_{AB}}=\frac{1}{1}+\frac{1}{KR}$$

The application of ATPS for recovery of biomolecules (pigments, enzymes, proteins, monoclonal antibodies) from plant cells and fermentation broths of diverse bacteria and yeast was recently reviewed [24].

In order to overcome a drawback of classical ATPS extraction (low density differences between phases causing slow phase separation or the need for centrifugation), Riedl and Raiser [31] proposed membrane supported liquid-liquid extraction (Memex) for recovery of biomolecules (Figure 1b). Memex requires two immiscible liquid phases, which are separated by the microporous membrane. However, the membrane itself does not prevent ingredients from going through it. On the contrary, when one of the two phases enter into the membrane pores, the phase contact occurs, and extraction takes place in the membrane pores. The stationary phase is saturated by target compounds, and the flow of mobile phase is co-current. By running the ATPS extraction in this direction, a decrease of the compound in one phase and an increase in the opposite phase are obtained. The mass transfer coefficient *K* describes the mass transfer of a component *i* from Liquid Phase 1 (wetting the membrane) to Liquid Phase *2*:

(3) $$\frac{1}{K_{i}}=\frac{1}{k_{1}}+\frac{1}{k_{Mem}}+\frac{V_{z}}{k_{2}}$$

where *k*_1_, transport resistance, m/s of component *i* in Liquid Phase 1, *k_Mem_,* transport resistance, m/s of component *i* in the membrane, *k*_2_, transport resistance, m/s of component *i* in Liquid Phase *2*, and *Vz*, mole fraction, c/c.

As it is advantageous if one phase fully wets the membrane and the other does not, the addition of surfactants is often required [32].

Another modification of ATPS is the aqueous two phase flotation (ATPF) system (Figure 1c). This concept combines solvent sublation with aqueous two phase extraction in which the surface active compound dissolved in water (salt phase) is adsorbed on the surfaces of gas bubbles in ascending stream and then collected in an organic layer (PEG) placed on top of the water column [33]. The concentration coefficient (α) of target compounds is described as follows:

(4) $$\alpha =\frac{C_{PEG}}{C_{wi}}$$

where *C_PEG_*, compound concentration in the PEG phase, and *C_wi_*, initial concentration of the compound in the aqueous phase, at time *t*.

The mass transfer in ATPF results in high separation performance and very low amounts of organic solvent needed. The volume of the aqueous phase is much bigger than the volume of the PEG phase; therefore, the concentration of ammonium sulfate is a significant factor allowing maintaining an immiscible two phase system via a salting out effect [33]. The first application of the solvent sublation extraction was the separation of penicillin G from fermentation broth. The solvent system was composed of an aqueous solution of ammonium sulfate (350 g/L), pH 6.8, and PEG solution containing 50% water (*v*/*v*) as the extraction phase. Under the optimal condition, penicillin G was obtained with an efficiency over 97%, a distribution ratio >100, and a concentration coefficient over 19 [33].

The two phase LLE of reaction products can also be performed with the application of aqueous-organic systems, ionic liquids, or natural deep eutectic solvents [26,34,35,36,37]. The enzymes’ activity and stability in these systems were confirmed, and interestingly applied solvents were even shown to enhance the production of desired target compounds [34,38].

#### 4.1.2. Natural Deep Eutectic Solvents

Natural deep eutectic solvents (NADES) were studied in the last decade as green media for extraction, with promising results. They are produced from plant based primary metabolites and composed of compounds such as choline, amines, sugars, polyalcohol, and carboxylic acids. NADES represents mixtures liquid at ambient temperature having unusual solvent properties [39]. These novel green solvents have advantages such as biodegradability, low toxicity, solute stabilization, sustainability, and low cost [40,41,42,43]. The pioneers in using deep eutectic solvents were Abbott et al. [44], who presented one of the first studies based on this new type of solvent, which were formed by mixing solid materials with high melting points. The NADES tend to be soluble in polar solvents, being insoluble in non-polar solvents [44]. Currently, deep eutectic solvents are used to extract polar and non-polar natural compounds [45]. NADES are proper for extracting different classes of active compounds, from different types of samples [46,47,48]. Depending on the chemical nature of their components, NADES can be classified into derivatives of organic acids, derivatives of choline chloride, mixtures of sugars, and others [49]. NADES can be obtained by the heating and stirring method [40], evaporation [40], or freeze-drying [50]. Recently, even the enantioselective reactions of the reduction of 1-(3,4-dimethylphenyl) ethanone and hydrolysis of (±)-1-phenylethyl acetate by carrot root in cholinium based eutectic mixtures as the solvent were reported [51]. However, it was shown that NADES cause the rupture of cellular membranes and leakage of enzymes; thus, they may facilitate the enzymatic reactions outside the cells and increase the yield, as demonstrated by Yang et al. [52].

#### 4.1.3. Counter Current Chromatography and Centrifugal Partition Chromatography

**Figure 2:**
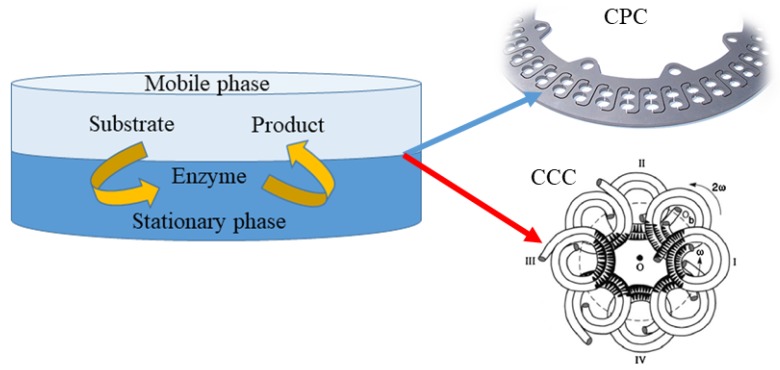
Schematic representation of approaches for the preparative isolation and purification of natural products using counter current chromatography (CCC) and centrifugal partition chromatography (CPC).

Other techniques being currently used for preparative isolation and purification of natural products are counter current chromatography (CCC) and centrifugal partition chromatography (CPC) (Figure 2). In both techniques, separation occurs between two immiscible phases (stationary and mobile), generating droplets or film and then coalescence. In CCC, the stationary phase is retained in the polytetrafluoroethylene coil by the gyratory motion, while in CPC, the constant gravity field produced by a single axis rotation, with rotary seals, is used to supply the stationary phase. For instance, Ma et al. [53] applied high speed counter current chromatography (HSCCC) for the purification of four prenylflavanones obtained in the microbial production of kurarione. A single run, stepwise elution with a two phase solvent system was composed of n-hexane-ethyl acetate-methanol-water at the volume ratios of 1:1:0.7:1 (*v*/*v*) and 1:1:1.2:1 (*v*/*v*) at 1.5 mL/min flow rate and 900 rpm revolution speed resulted in 56% retention of the stationary phase and yielded 4α,5α-dihydroxy norkurarinone, 7-methoxyl-4α,5α-dihydroxy norkurarinone, 6α-hydroxykurarinone, and norkurarinone with purities over 94%. Further, Xia et al. [54] elaborated the efficient HSCCC separation procedure for theaflavins obtained in the tannase mediated product (TBP). The maximum sample loading enabled obtaining theaflavin, epigallocatechin, and epicatechin with purity over 96%. Furthermore, the laccases, copper containing oxidase enzymes, were purified from a fermentation broth of *Pleurotus sapidus* with CPC in an aqueous two phase system composed of PEG-3000 solution (13% *w*/*w*) and phosphate buffer at a pH value of 7, with 2.5% (*w*/*w*) addition of NaCl [55]. The total activity of investigated enzymes was preserved in the suitable solvent system and mild operating conditions, confirming the suitability of CPC for bioactive compounds’ separation.

#### 4.1.4. Ultrasonic Assisted Extraction

The ultrasonic assisted extraction (UAE) is also suitable for the recovery of natural products, removing the possibility of the nonthermal decomposition of heat sensitive compounds [56]. The repeated compression/expansion cycles caused by ultrasonic waves create cavities in the liquid. At the same time, the waves act through swelling/hydration processes, for the enlargement of the cell wall pores, thus improving the diffusion without the disruption of cell wall/membranes. Hence, phase transfer goes from the disperse phase into a continuous phase, which with higher vapor pressure, lower surface tension, and lower viscosity tends to easily form the liquid cavities [57]. UAE decreases the processing time and volume of the solvent used, at the same time increasing the extraction yield. However, the extraction yield has to compromise the prevention of cells’ integrity and enzyme/cells’ activity, which is obtained at low temperatures and with biocompatible solvents [58]. The UAE was proven to be a biocompatible extraction method to obtain rosmarinic acid (RA) from mycorrhizal hairy roots of basil (*Ocimum basilicum* L.). The application of 10% aqueous methanol with 15 min sonication at a frequency of 45 kHz preserved roots and mycorrhizal viability and resulted in a very good yield of product compound [58]. In another study, Jiao et al. [59] successfully applied UAE for the extraction of isoflavonoids from milkvetch (*Astragalus membranaceus* (Fisch.) Bunge) hairy root cultures.

#### 4.1.5. Supercritical Fluid Extraction

The application of supercritical fluid extraction (SFE) for processing of biotechnological production is not new. It allows the removal of organic solvents form the extraction process. The principle of this technique is based on the use of supercritical fluid, usually CO_2_ in the supercritical state (temperature above 31.1 °C and pressure above 72.8 bar), which has both gas and liquid properties, also being a solvent Generally Recognized as Safe (GRAS) [60]. The low viscosity and high diffusivity of CO_2_ improve the mass transfer and reduce the extraction time. By manipulating the solvent density and by adding co-solvents, high selectivity can be obtained [61]. The SFE technique allowed the selective extraction of sulfur containing compounds from lyophilized hairy roots of genetically transformed French marigold (*Tagetes patula* L.). Four characteristic thiophene metabolites were obtained in 60 min dynamic time with scCO_2_, at 30 MPa and 40 °C. Application of methanol as a co-solvent proved not to increase the selectivity [62]. Later, Anuar et al. [63] compared SFE with classical solvent extraction for the recovery of quercetin and (+)-catechin from gale of the wind (*Phyllanthus niruri* L.) callus cultures. The content of quercetin and (+)-catechin was respectively up to 2.1 and up to 11.7 fold higher in scCO_2_ SFE than in conventional extraction. The addition of a modifier (ethanol) was also an important factor in this case, improving the extraction efficiency [63].

#### 4.1.6. Supercritical Reactive Extraction

Another industrially relevant application of SFE for recovery of biomolecules from fermentation broth is a combination of this technique with reactive extraction (supercritical reactive extraction). The process of reactive extraction involves the use of specific extractants, mixed extractants, extractants mixed with conventional solvents (diluents), or active diluents with active functional groups [64]. In the extraction of polar compounds using non-polar scCO_2_, the use of extractants forming complexes is required. The extractants additionally increase the solubility of the extracted compounds in supercritical fluid [65]. In many cases, the addition of diluents is necessary because of the extractant’s high viscosity and caustic character. The diluents control the physical properties such as the density, viscosity, and surface tension of the organic phase [66]. The long chain aliphatic tertiary amines (e.g., trioctylamine) combined with scCO_2_ can be successfully used for reactive extraction of citric acid from aqueous solutions. The comparison of semi-continuous and batch mode revealed that the first one leads to a higher efficiency of citric acid separation due to the avoidance of the effect of low trioctylamine solubility in scCO_2_. The factors such as pressure and temperature influenced the final reactive extraction efficiency, which was the best (96.9%) with high pressure (16 MPa) and low temperature (35 °C) using a flow of scCO_2_ saturated with trioctylamine [65]. The supercritical extraction is a promising technique when reactive extraction is needed, with particular application to the extraction of carboxylic acids from fermentation broths [64,67].

### 4.2. The Recovery of Metabolites from Disrupted Cells

#### 4.2.1. Microwave Assisted Extraction

In classical processing of natural products obtained in cell/organ cultures, the additional step of water removal from biomass is required. The dry biomass is usually obtained via lyophilization and subsequently subjected to conventional reflux extraction, MAE, or UAE [68,69].

Microwave assisted extraction (MAE) is successfully applied to destroy cell/culture matrices, hence to release produced compounds, which diffuse out and dissolve in extraction solvents. MAE involves the dissipation of electromagnetic waves in the medium, causing vibration of water and other polar molecules [70]. This results in an increased temperature in the intracellular liquids, subsequent evaporation of water, and accumulation of pressure in cells. Besides rupture of cells, the disruption of hydrogen bonds is observed, leading to the migration of dissolved ions and increased penetration of solvent into the sample [71]. The microwaves cause local heating within the sample with the rapid increase of the temperature, facilitating the diffusion of target molecules, with decreased amounts of solvents and time and energy consumption [72]. MAE has higher performance when operating under atmospheric conditions [73]. The advantage of dynamic MAE over dynamic solvent extraction without microwave assistance when applied to dried callus tissue was presented by Gao et al. [74]. Another modification of MAE was presented by Lee and Kim [75], who proposed the recovery of paclitaxel from plant cell cultures and simultaneous removal of plant derived tar and waxy compounds. The biomass mixed with adsorbent was subjected to MAE and subsequent LLE and hexane precipitation, enabling over 99% recovery of paclitaxel with a purity of 22%. The result in yield and purity was comparable with the conventional paclitaxel separation/purification procedure involving the additional step of adsorbent treatment. Thus, the application of simultaneous MAE and adsorbent treatment instead of classical solvent extraction and subsequent adsorption can be advantageous for reducing the production costs [75].

In order to eliminate the step of biomass drying, homogenization coupled to extraction can be applied. High speed homogenization (HSH) is an effective sample pretreatment technique facilitating the disruption of cell/organ cultures and thus the release of their content into the liquid environment. HSH involves strong shear and thrust forces generated by high rotation speed (10,000, 20,000 rpm), which causes the grinding of sample particles. The pulverization of the hydrated sample in a continuous slurry enables avoiding the localized increase of temperatures, thus reducing the risk of degradation of the thermolabile compounds [76].

HSH coupled to MAE provides higher homogenization efficacy, as under microwave irradiation, the in situ water in the cell/organ cultures rotates, and immediate internal changes, leading to an additional breakdown of cell walls/membranes and exhaustive release of intracellular target molecules. The water mediated heating can effectively deactivate hydrolytic enzymes and thus reduce or prevent the degradation of the product during extraction [77,78]. The first report about the application of HSH coupled MAE for direct extraction of phytochemicals from fresh plant in vitro cultures was described by Jiao et al. [77]. The yield of alkaloids and flavonoids extracted from woad (*Isatis tinctoria* L.) hairy root cultures was higher comparing with Soxhlet extraction (SE) and UAE, while the extraction time, energy cost, and CO_2_ generation were reduced significantly. The cytohistological studies confirmed evident rupture of cellular matrices, being a result of pressure buildup within cells [77].

#### 4.2.2. Ultrasound Assisted Extraction

A similar highly effective cell disruption in fresh biomass can be obtained via coupling HSH with cavitation accelerated extraction (CAE) [78]. CAE causes cavitation by negative pressure generated via a vacuum pump. When the air is introduced into a liquid-solid system and the bubbles are collapsing, the intensive cavitation-collision, turbulence, suspension, and interface effects are produced, enhancing disruption of cells and accelerating mass transfer between the extraction solvent and matrix. CAE causes cavitation of much lower intensity than UAE and maintains a constant low temperature, thus preventing the degradation of thermosensitive compounds [79,80]. The coupling of HSH with CAE eliminates biomass drying and grinding. In comparison to SE and UAE, HSH-CAE provides the highest efficiency and yield. At the same time, it lowers energy cost and CO_2_ generation [78].

### 4.3. Simultaneous (Bio)Conversion and Separation with Application of Counter Current Chromatography

The simultaneous production and separation of the final product can be obtained via immobilization of the enzyme/microorganism in the liquid stationary phase based on the high affinity and partitioning of the substrate to the mobile phase passing through the column. The described mechanism providing the reaction zones of mixing and separation of a product from the substrate can be obtained using multi-phase reaction systems: CCC and CPC. The concept of the utilization of CCC and CPC as enzymatic reactors is not new; however, these techniques are continuously improved for efficient production and separation of target compounds [81,82,83,84]. When the product is removed from the catalytically active phase, the reaction equilibrium is shifted, resulting in high product yield [85]. Originally, aqueous two phase systems composed of water and either two immiscible polymers or a polymer and a salt, which above a critical concentration, makes two immiscible phases, were used for the purification of biomolecules, cellular organelles, or whole cells. Recently, aqueous-organic two phase system for the conversion of hydrophobic substrates in CPC and high speed counter current chromatography (HSCCC) reactors were proposed [82,83,84,85,86]. Furthermore, ionic liquids having the ability to dissolve many compounds and form two phase systems were successfully utilized for biocatalytic reactors in CCC [87,88]. The application of these techniques requires the selection of suitable enzyme by optimization of enzyme kinetics parameters, determination of partition coefficients for the enzyme, substrate, and product, determination of enzyme activity in the selected two phase system, and optimization of the flow rate and rotation speed.

CPC chromatography combining bioconversion and separation was shown to provide competitive mixing conditions compared to the stirred tank reactor (STR) when the reaction performance was dependent on the mass transfer on the interfacial layer. What is more, giving a good distribution of reaction volume into the chambers, the CPC could operate low volumes of aqueous phases more efficiently than STR [81,85]. However, the efficiency of the CPC reactor is restricted by the interface renewal rates and interface residence time at higher enzyme levels. Therefore, the increase of the chamber size and development of CPC rotors or chamber geometries are needed for bigger scale applications [81]. More efficient bioreactors were recently designed applying HSCCC separation. This type of immobilized enzyme reactor implies simple operation and the ability to recycle the enzyme in the aqueous organic two phase system. Applying this technique, Song et al. [82] performed the enzymatic transformation of polydatin to resveratrol. Furthermore, Wang et al. [83] applied an HSCCC reactor for bioconversion of soybean isoflavone glucoside conjugates (accounting for more than 95% of the total isoflavones) to aglycones, having higher absorption rates in the intestine. The bioconversion reactions of plant secondary metabolites (PSMs) performed in CCC reactors proved that after optimization, CCC chromatography could be successfully applied in one step substrate transformation and product purification.

## 5. Advanced Extractions Techniques for Obtaining Biologically Active Compounds from MAP

### 5.1. Classical and Modern Extraction Techniques

Classical techniques are based on solid-liquid extraction with various solvents, having significant drawbacks, both in terms of long extraction time and of large amounts of organic solvents used. As an alternative, the development of efficient and cost effective downstream processes represented the focus of recent research. Among other parameters, the selection of an appropriate solvent is of utmost importance and strongly defines the yield and composition of the extracts produced. Extraction techniques and their utilization in analytical and pharmaceutical fields have been greatly developed due to the progress of the modern instruments, these being used in their simple variants or in tandem with other equipment (Table 2).

Due to low bioactive compounds extraction yields, traditional extraction techniques might eventually be replaced with modern techniques: microwave or ultrasound assisted extraction, pressurized liquid extraction, liquid-liquid micro-extraction, techniques based on eutectic solvents or enzymatic treatment, etc.

According to Handa [89], maceration, percolation, and infusion are the general techniques used for the extraction of medicinal plants applied for galenical preparations. Usually, water is used as the solvent, but the process can also be performed in alcoholic solutions to obtain alcoholic macerates [94] (Table 3).

Another classical extraction technique is Soxhlet extraction (SXE), which combines the benefits of the reflux extraction with percolation processes. The extraction techniques in variants of automated equipment are able to minimize the disadvantages and improve the performance, such as shortening leaching times [95].

The used solvent is an important factor for the extraction technique, alongside other parameters, such as the temperature of the system, the ratio of the sample, and the chemical and physical properties of the sample intended for extraction [96]. One disadvantage of the classical solid-liquid techniques (besides the enhanced weight of raw plant material, the large amount of used solvent, energy and time consumption) is the large amount of plant material remaining after the process, which is still rich in bioactive compounds that have not been extracted. These methods are also not suitable for phytoconstituents with the risk of thermal degradation [97].

#### 5.1.1. Microwave Assisted Extraction

**Figure 3:**
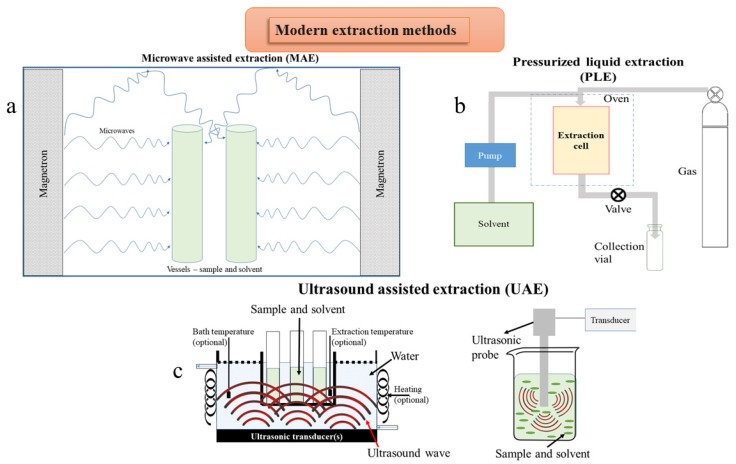
Schematic representation of modern extraction techniques: (**a**) microwave assisted extraction; (**b**) pressurized liquid extraction; (**c**) two setups for ultrasound assisted extraction.

Low energy-high efficiency extraction is based on processes with reduced energy consumption, decreased quantity of raw material, and increased yield of the final biologically active compounds. The technologies used to optimize the production of bioactive compounds have been developed in the last decades. The modern techniques can be classified into three categories: (1) based on microwave power (ME), (2) pressurized liquid extraction (PLE), subcritical water extraction (SWE) and supercritical fluids extraction (SFE), and (3) ultrasound assisted extraction (UAE) (Figure 3, Table 4). These techniques can be mainly used to extract active compounds on an industrial scale, having at the same time several “green” characteristics (shorter extraction time, no use of toxic chemicals, higher extraction yields with low solvent and energy consumption). Several alternatives to the use of classical organic solvents emerged in the last few decades (ionic liquids, agro-solvents, like alcohols or terpenes, or the use of surfactant solutions). The extracting selectivity can be modulated by using mixtures of solvents. Solutions of ionic liquids (ILs) are suitable solvents for the MAE of PCs from medicinal plants [99].

By optimizing the energy absorption and reducing the temperature fluctuations in the microwave technique, extraction efficiency and extract quality can be increased [100,101]. The traditional extraction of essential oils (EO) and non-volatile components involved individual, time consuming, and inefficient procedures. Liu et al. [102] used the ionic liquid mediated microwave assisted hydro-distillation concatenated liquid-liquid extraction (ILMHDE) technique to extract EOs rich in astragalin, quercetin, luteolin, kaempferol, and apigenin from immortelle (*Helichrysum arenarium* (L.) Moench). By this technique, small amounts of essential oil components that often form a hydrosol with condensate water can be separated and collected. Compared with the traditional technique of heat reflux extraction (HRE) or microwave hydro-distillation, ionic liquid based microwave assisted simultaneous extraction and distillation (ILMSED) can produce higher yields of essential oils rich in phenolic acids [103], together with simultaneous extraction of non-volatile compounds. Using ionic liquids as extraction solvents, cell walls are affected, leading to enhanced yields of active compounds in the extraction solution. Microwave assisted simultaneous distillation extraction (MASDE) can be used for the simultaneous extraction of active compounds with the distillation of EOs [104]. Dual cooled solvent-free microwave extraction (SFME) can be used with a modified Clevenger-type apparatus to obtain essential oils [105]. Furthermore, NADES can be used as a solvent for the MAE technique; the factors that influence the process are sample/solvent ratio, time and temperature of extraction, and NADES dilution and composition [105,106].

This technique is considered a green one, due to the minimized solvent amounts, thus reducing waste production or the emission of CO_2_, including hydrotropic liquids, two phase solutions, miscellas, etc. The transformation of electrical energy into thermal energy is expressed as [107]:

(5) $$P=K.f\varepsilon^{\prime }E^{2}tan\delta$$

where *tan δ*, dielectric loss tangent, *E*, electric field strength, *ε*′, dielectric constant, *ε*″, efficiency of transforming microwave irradiation into heat, *f*, the frequency used, *K*, constant, and *P*, microwave power dissipation per unit volume, where the absorbed energy is derived from the dissipation factor (*δ*) equation:

(6) $$tan\delta =\frac{\varepsilon^{\prime \prime }}{\varepsilon^{\prime }}$$

The MAE system can also operate under nitrogen protection to avoid the oxidation of the obtained compounds [108].

#### 5.1.2. Pressurized Liquid Extraction

Pressurized liquid extraction (PLE) includes all techniques that perform the extraction under pressure, such as accelerated solvent extraction (ASE), enhanced solvent extraction (ESE), or high pressure solvent extraction (HSPE) [109]. The process involves the solvent to be at elevated temperatures and pressures, in order to extract analytes from solid samples. The most important parameters for this technique are the polarity of the extraction solvent, temperature, and pressure, which control the process mechanisms and the number of extraction cycles [110]. The enhanced yield of the extraction is due to the elevated temperature, which increases the solubility of the compounds, and to the solvent, which is below its boiling point due to the high pressure.

Accelerated solvent extraction (ASE) offers many advantages: good reproducibility and shorter extraction time, the possibility to adjust extraction temperature, and purification of extracts on-line [92]. In order to obtain extracts with enhanced active compound concentrations responsible for obtaining metallic nanoparticles, Fierascu et al. [111] used accelerated solvent extraction for leaves of certified lemon balm (*Melissa officinalis* L.).

If water is the applied solvent, then the technique is called pressurized hot water extraction (PHWE). In this case, the phenomenon of self-ionization of water appears, which respects the following equation [112]:

(7) $$K_{w}=\frac{\left[H_{3}O^{+}\right]\left[OH^{-}\right]}{{\left[H_{2}O\right]}^{2}}$$

The PHWE is useful for extraction of non-polar, moderately polar, and polar compounds [113]. This technique is a good alternative to hydro-distillation (HD), steam distillation, or organic solvent methods, for the separation of compounds like verbenone, germacrene D, bornyl acetate, ferruginol, trans-caryophyllene, elemol, γ-cadinene, geraniol, or β-eudesmol [98]. ASE can be used to obtain extracts enriched in polyphenolic compounds, as demonstrated by Gomes et al. [114] on different species of passion flowers (*Passiflora sp.)*. The performance of three different extraction procedures was presented by Herrero et al. [115]. The authors compared PLE, using water and ethanol as solvents, SFE, and using CO_2_ and supercritical CO_2_ modified with ethanol or water and particle formation on-line (WEPO) in order to obtain increased yields of carnosic and rosmarinic acids from rosemary (*Rosmarinus officinalis* L.). Subcritical water extraction (SWE) represents a modern alternative for the extraction of biologically active compounds, in which the temperature represents an important parameter that must be optimized [116], together with the extraction pressure. Water at high temperature is an effective solvent for both polar and non-polar compounds, the dielectric constant of water becoming similar to that of an organic solvent. Tuning the dielectric constant of water results in increased yields of bioactive compounds [117]. Besides this, if organic and inorganic modifiers are added, the extraction yield can be further enhanced [118].

#### 5.1.3. Ultrasound Assisted Extraction

In the case of ultrasound assisted extraction (UAE), the solvents can be water, ionic liquids, ethylene glycol and its oligomers, glycerol, or other biomass based solvents [119,120]. Ultrasonic assisted enzymatic extraction (UAEE) depends on reaction time, the type and concentration of enzyme, temperature, pH value, and the particle size of plant material [121]. This technique can be performed with less energy consumption, at lower temperature, in a short period of time. The cells of plant material can be destroyed by hydrolytic enzymes. Being a proper method, it can be used to extract polysaccharides from different materials [122], the release of the active compounds being obtained through the enzyme degraded cell walls [123]. Polysaccharides were obtained from bitter melon (*Momordica charantia* L.) [124] or Japanese knotweed (*Polygonum cuspidatum* Siebold & Zucc.) [125]. Conventional techniques have increased yield of extraction, but enhancement of the extracted compounds can be done through biotechnological methods, like the addition of specific enzymes (cellulase, α-amylase, pectinase) during extraction [126]. Enzyme assisted extraction (EAE) can be divided into enzyme assisted aqueous extraction (EAAE) and enzyme assisted cold pressing (EACP), both eco-friendly technologies for the extraction of bioactive compounds and oils, replacing organic solvents with water [127,128].

### 5.2. Other Extraction Techniques

#### 5.2.1. Ohmic Assisted Technologies

Ohmic assisted technologies are those techniques relying on ohmic heating by passing an electrical current through materials, instead of conductive heat transfer. Initially, this technique was used for other applications, like fermentation or sterilization, but it was proven to be suitable for the extraction of active compounds [130]. Due to the conversion of electrical energy to thermal energy within the matrix, the parameters involved in this technique are: electrical conductivity of the materials (plant and solvent), input energy, and electrodes. In ohmic assisted hydro-distillation (OAHD), the traditional heating source is replaced by ohmic heating, and Ohm’s law, the power equation, and Joule’s first law are respected [131].

(8) $$I=\frac{V}{R}$$

(9) P=I×V

(10) $$P=\frac{V^{2}}{R}$$

(11) $$q=\sigma \times E^{2}$$

where *I*, electric current (measured in Amperes), *V*, voltage (in Volts), *R*, resistance (Ohm), *P*, electric power (Watt), *q*, internal energy generation rate (W/m^3^), *σ*, electrical conductivity of the material (Siemens/m), and *E*, electric field strength (V/m).

Therefore, the important parameters for this technique are the electrolyte concentration, the use of pretreatment in the sample preparation stage, and the voltage and frequency of the current [132].

The heating of plant material and solvent are beneficial in releasing bioactive compounds; thus, this process is a technique with a short processing time. The technique is an environmentally friendly one, but the disadvantages in applications are related to the maintenance of electrodes and the necessity of using non-electroconductive materials for the containers or heating chamber used.

Due to the increased yields of extraction, OAHD can be considered a technique with significant industrial relevance, being more efficient than conventional techniques (HD) [133]. This method was applied to obtain increased yields of essential oil from peppermint (*Mentha piperita* L.) and oregano (*Origanum vulgare* subsp. *viride)* [132,133] (Table 5).

#### 5.2.2. High Voltage Electric Discharge

High voltage electric discharge (HVED) is an emerging technology commonly used in chemical removal of organic impurities present in water [134], currently being used in the food industry [135]. This technique has the advantages offered by the control of voltage: enhancing heat and mass transfer with their control and applicability to single phase and multiphase flows. The parameters that can be modified are the voltage, electrode geometry, shape of pulses, and mode of actions [4]. Corona discharge is a high voltage phenomenon produced between at least two electrodes, the corona electrode (a sharp electrode) and a blunt electrode with a larger radius (grounded electrode).

For this phenomenon, the following equation is respected [136]:

(12) $$F=\rho_{e}E+\frac{\varepsilon_{0}}{2\left[\nabla \left(\frac{E^{2}\rho_{g}dk}{d\rho_{g}}\right)-E^{2}\nabla k\right]}$$

where *ρ_e_*, electric charge density, *E*, electric field vector, *ε*_0_, permittivity of free space, *k*, dielectric constant of the fluid, and *ρ_g_*, fluid mass density.

The corona discharge converts electrical into mechanical energy:

(13) $$v={\left(\frac{\varepsilon_{0}}{\rho_{g}}\right)}^{\frac{1}{2}}E$$

where *υ*, ionic wind velocity.

The corona discharge occurs only when a certain voltage level is exceeded; with the increase of the voltage, the ionization process becomes more intense and leads to a more important electric current.

In the case of the extraction of bioactive compounds, the process can be enhanced by pulsed high voltage electrical discharges in water or liquid medium. Application of a high voltage excites fluids’ molecules, obtaining a stream of electrons, which emit photons; the photons can ionize nearby atoms, creating free electrons, resulting in a chain reaction. The bubbles obtained from the dissociation of liquid or local vaporization through electron stream can produce cell disruption, particle fragmentation, and cell structure damage, with mass transfer processes for the extraction of biomolecules [136].

This technique is considered a promising green extraction method for promoting cell disruption and enhancing the extraction yield of different compounds: protein and phenolic compounds from the olive kernel (*Olea europaea* L.) [137], oil from sesame seeds (*Sesamum indicum* L.) [138].

#### 5.2.3. Pulsed Electric Field

Pulsed electric field (PEF) uses short pulses of electricity (from μs to ms) under high intensity electric fields (kV/cm), which leads to the formation of pores on the cell membranes with improving the extraction and diffusion processes, causing the permeabilization of the cell membrane. This method ensures non-thermal permeabilization of cellular membranes and prevents the cell walls from undergoing thermal alteration. Using this technique as an extraction or as a pretreatment method, enhanced yields of bioactive compounds were obtained, such as betanin from beetroots (*Beta vulgaris* L., 1753) [139]; for maize (*Zea mays* L.), the recovery of phytosterols was increased by 32.4% using PFE as a pretreatment [140]; or improved antimicrobial effectiveness for different extracts was observed [141].

## 6. Experimental Design of Phytoconstituents’ Production and Recovery

Experimental design plays an important role in the optimization of extraction procedures, particularly in industry, but also in science. The concept of mathematical modeling is based on the optimization of the factors influencing the response in order to obtain response values according to the predicted value. Statistical design of experiments (DoE) is a simpler and conventional statistical tool, with a more systematic experimental design, which can offer significative results after implementing its three steps: design, analysis, and prediction phases. It is usually used for the determination of phenolic compounds from vegetal material [142]. The response surface methodology (RSM) represents an important statistical tool for the optimization of the independent factors influencing the response of a set of experiments and is one of the DOE relevant multivariate techniques. The method involves the variation of a single factor at a time, with the others kept constant, representing a one-factor-at-a-time approach. RSM helps to decrease the numbers of experimental runs to a minimal number required by the experimental model in a full factorial design [143]. It can be run after identification of the problem, determination of the responses, identification of extraction variables, selection of the proper design, evaluation of the model, optimization, and validation of the model. RSM is usually applied to design experiments with more than two variables interacting with one or more response variables. The interactions can be evaluated in Box-Behnken designs (BBD), central composite design (CCD), central composite rotatable design (CCD), or face-centered cubic experimental design (FCD). The first two experimental designs are more frequently used, especially to develop the methods at a large scale [144]. RSM is successfully utilized in biotechnological approaches, providing information regarding the production of PSMs, or extraction techniques for active compounds from plants materials (Table 6).

The artificial neural network (ANN) model falls under the broad category of artificial intelligence. Compared to RSM, ANN is an excellent statistical tool, due to its ability to learn from observations, conclude by generalizing, and predictive modeling of complex non-linear processes [148]. This method was successfully applied for different extraction techniques in order to obtain active compounds, such as microwave assisted extraction of candy leaf (*Stevia rebaudiana* Bertoni) leaves [148] or to different solvents used in order to indicate how the recovery percentage of active compound could be optimized [151]. A novel ANN-RSM combined approach was developed to predict the variation of hyoscyamine content and to explore statistically the relationship between the response and the factors involved in the experiment of Amdoun et al. [144]. Their model was applied to the response of jimsonweed (*Datura stramonium* L.) hairy roots elicited by salicylic acid.

## 7. Future Directions

The trends of obtaining bioactive compounds have changed in the last few decades. In this context, the search for innovative approaches to obtain phytoconstituents from medicinal and aromatic plants is highly relevant.

The examples of the application of PCTOC for large scale production of biomass and therapeutic molecules clearly outlines their immense potential for upstream plant biotechnologies for future wider industrialization. However, several challenges are still to be addressed, mostly related to the relatively high sensitivity of plant cells to different stress factors/conditions and hence better understanding of the cellular/molecular responses to the bioreactor environment. The influence of the cultivation conditions in bioreactors was investigated mainly with respect to single compound bioproduction (e.g., rosmarinic acid, for instance), while the impact on the whole metabolic network offers a wide range of research for the future. As future perspectives, further optimization should be applied to decrease the overall time necessary for the batch processes (such as filling up, emptying, cleaning, and sterilization of the bioreactor system), in order to increase the efficiency of the processes at the industrial level.

The development of efficient and cost-effective downstream processes represents the main challenge. Among many modern techniques providing very good extraction performance, electro technologies (high voltage electric discharge, pulsed electric field, ohmic assisted extraction) seem to be promising techniques for direct recovery of plant bioactive compounds. Future perspectives are going for decreasing maintenance costs of these equipment, in order to be economically feasible and competitive.

In order to enhance the competitiveness of different industries, the development and adaptation of the abovementioned methods at industrial levels needs to respond to several questions: (a) Is a good strategy applied to obtain specific and selective phytoconstituents from different matrixes? (b) Is the valorization of plant material economically feasible when scaled-up?

Although there is an abundance of literature and information regarding the obtaining of phytoconstituents from vegetal material at the laboratory scale, further studies are needed in order to scale-up the applications, in a cost and resource effective manner and with a low impact on the environment.

## 8. Conclusions

In the present critical review, aspects regarding plant biotechnology were discussed, which permit the production of plants with increased levels of fine chemicals and greener downstream processing methods, in order to obtain bioactive compounds from both fresh plant materials and the remaining by-products. The trend of science presented in this paper confirmed the prediction that the increased interest in the compounds obtained from plants will lead to the development of new methods and techniques or will improve the existing ones.

## Figures and Tables

**Table 1:** Definitions of the terms and techniques used.

Term	Definition
ANN	Artificial neural network, computational modelling technique, based on simulations of the biological brain, with which can be drawn conclusions through generalization
AFE	Accelerated fluid extraction, extraction method based on solvent with high pressure and temperature
CAE	Cavitation accelerated extraction, extraction method based on occurring cavitation due to the passage of ultrasound waves in the liquid medium
CCC	Counter-current chromatography, chromatographic techniques used for preparative isolation and purification of natural products
CPC	Centrifugal partition chromatography, chromatographic techniques used for preparative isolation and purification of natural products
EAAE	Enzyme assisted aqueous extraction, extraction method enzyme assisted in aqueous medium
EACP	Enzyme assisted cold pressing, extraction method enzyme assisted, used especially for obtaining oils
GAE	Gallic acid equivalents
HVED	High voltage electric discharge, non-conventional extracting method based on corona discharge, which through the control of voltage, can enhance heat and mass transfer; the process can be enhanced by pulsed high voltage electrical discharges in water or liquid medium
ILMHDE	Microwave assisted hydro distillation concatenated liquid-liquid extraction, extraction technique based on microwave concatenated with a liquid-liquid extraction installation, with two columns, which can separate essential oils (in the first separation column) and extract some components from the hydrosol (in the second separation column)
ILMSED	Ionic liquid based microwave assisted simultaneous extraction and distillation, microwave assisted simultaneous extraction and distillation, which use as solvents ionic liquids
LLE	Liquid-liquid extraction, extraction method based on the relative solubilities of compounds in two different immiscible liquids
Low energy-high efficiency extraction	Extracting processes with reduced energy consumption, decreased quantity of raw material, and increased yield of the final biologically active compounds
MAE	Microwave assisted extraction, extraction method based on microwave power with damaging of cells’ structure
MASDE	Microwave assisted simultaneous distillation extraction, extraction method with two simultaneous processes, microwave assisted extraction and the distillation of essential oils
NADES	Natural deep eutectic solvents, solvents based on more compounds produced from plant based primary metabolites liquid at ambient temperature having unusual solvent properties
OAHD	Ohmic assisted extraction, nonconventional extracting method that relies on ohmic heating by passing an electrical current through materials, instead of conductive heat transfer
PEF	Pulsed electric field, extraction techniques that use short pulses of electricity (from μs to ms) under high intensity electric fields (kV/cm), which leads to the formation of pores on the cell membranes with improving the extraction and diffusion processes, causing the permeabilization of the cell membrane
PHWE	Pressurized hot water extraction, techniques that perform the extraction under pressure, with water as a solvent
PLE	Pressurized liquid extraction, extraction method that performs the extraction under pressure
RSM	Response surface methodology, statistical methods used for process optimization and modelling through empirical models
SFE	Supercritical fluid extraction, extraction method that performs the extraction under pressure with CO_2_ as a solvent
SFME	Solvent-free microwave extraction, modified Clevenger-type equipment to obtain essential oils based on microwave
SLE	Solid-liquid extraction, a multi-step extraction technique, resulting in the release of the target product with or without disruption of the cells with a counter current operation
UAE	Ultrasonic assisted extraction, extraction method based on repeated compression/expansion cycles caused by ultrasonic waves without the disruption of cell wall/membranes
UAEE	Ultrasonic assisted enzymatic extraction, extraction method based simultaneous on ultrasound and enzymatic treatment
WEPO	Water extraction and particle formation on-line, extraction method that performs the extraction under pressure with a mixture of CO_2_ and ethanol or water and particles as a solvent with formation on-line

**Table 2:** Comparison of different conventional extraction techniques.

Extraction Technique	Advantages	Disadvantages	Ref.
**Classical techniques**	- Large amounts of biomass	- High amounts of solvents - Low yield of bioactive compounds - Consumes energy - Plant wastes still found in active compounds	[89]
**Pressurized liquid extraction**			
Supercritical fluid extraction	- Increased selectivity - Avoid sample oxidization in the presence of air - Small amounts of solvents	- Expensive equipment - Extra cost for fluids	[90]
Subcritical fluid extraction	- Increased selectivity - Green solvent	- Expensive equipment	[91]
Accelerated solvent extraction	- Decreased time consumption and solvent use	- Need for high temperature and pressure - Expensive equipment	[92]
**Microwave assisted**	- Increased amounts of solvent - Increased extraction time - microwaves are non-contact heat source - microwaves can selectively heat the materials	- Expensive equipment	[93]
**Ultrasound assisted**	- Increased amounts of solvent - Can be used as pre-treatment for other techniques, increasing the yield of extraction - Breaking the walls of cells	- Good optimization of the parameters	[57]

**Table 3:** Classical extraction methods applied to different plants materials and the obtained compounds (in chronological order of the cited works).

Plant Material	Family Name	Extraction Method	Plant Organ	Conditions	Obtained Compounds	Ref.
Thyme, *Thymus vulgaris L.* Sage, *Salvia officinalis L.* Marjoram, *Origanum majorana L.*	Lamiaceae	Maceration	Leaves	Room temperature Time (hour), 72 Solvents: methanol, ethanol, diethyl ether, and hexane	Caffeic acid, p-coumaric acid, ferulic acid, cinnamic acid (hydroxycinnamic acids) carnosic acid (diterpene), rosmarinic acid (caffeic acid ester), apigenin (flavone)	[96]
Chamomile, *Matricaria recutita* L. Coriander, *Coriandrum sativum* L. Liquorice, *Glycyrrhiza glabra* L. Southern blue gum, *Eucalyptus globulus* Labill.	Asteraceae Apiaceae Fabaceae Myrtaceae	Alcoholic maceration	Flowers Seeds Roots Leaves	Solvent: grape marc distillates, alcohol content (*v*/*v*), 70%, 55%, 40%. Concentration of plants in the macerate (g/L) 40, 25, 10	Terpenes	[94]
Rosemary, *Rosmarinus officinalis* L., Immortelle, *Helichrysum italicum* (Roth) G. Don fil.	Lamiaceae Asteraceae	Hydrodistillation	Leaves and flower	500 kg of dried plant Time (hour), 3 Without any sample pre-treatment	Monoterpenoids	[98]

**Table 4:** Modern extraction methods applied to different plants material and the obtained compounds (in chronological order of the cited works).

Plant Material	Family Name	Extraction Method	Plant Organ	Conditions	Obtained Compounds	Extraction Yields (Content)	Ref.
Guava, *Psidium guajava* Linn. China root, *Smilax china* L.	Myrtaceae Smilacaceae	ILs-MAE	Tubers	1.0 g of accurately weighed sample, extracted with 20 mL of different ILs’ solution; extraction time (min) 10 at 70 °C; extraction time (min) 10 at 60 °C	Gallic acid, ellagic acid, (phenolic acids), quercetin (flavonol), trans-resveratrol (stilbenoid)	79.5–93.8%	[99]
Rosemary, *Rosmarinus officinalis* L.	Lamiaceae	PLE SFE WEPO	Leaves	**PLE:** Temperature (°C), 50, 100, 150, 200; static extraction time (min), 20; warming, up time (min), 5,7, 9 **SFE:** Temperature (°C), 40; pressure (bar), 150; CO_2_ flow rate (g/min), 60 **WEPO:** Temperature (°C) = 200; flow rate of supercritical water (mL/min), 0.2	Carnosic (diterpene) and rosmarinic acids (caffeic acid ester)	17.8–37.9%/ 0.5–6.5%/4%	[115]
Rosemary, *Rosmarinus officinalis* L.	Lamiaceae	ILMSED	Fresh leaves	Microwave irradiation frequency (GHz), 2.45; power (W), 120–700	Carnosic (diterpene), rosmarinic acids (caffeic acid ester), and essential oil	0.49–33.29%/0.07–3.97%/18.5–23.1%	[103]
Ginkgo, *Ginkgo biloba* Linn.	Ginkgoaceae	EAE	Leaves	time (hours), 30; ethanol-water ratio of 3:7 (*v*/*v*); pH 6	Flavonoids	14–28.3%	[127]
Cinnamon, *Cinnamomum verum* J. Presl	Lauraceae	ILMSED	Inner bark	Microwave irradiation frequency (GHz), 2.45; power (W), 120–700	Proanthocyanidins and essential oil	1.24–4.58%	[104]
Lemon balm, *Melissa officinalis* L.	Lamiaceae	PLE	Leaves	time (min), 10; temperature (°C), 150 °C; extracting time (min), 20; warming-up time (min), 7	Rosmarinic acid (caffeic acid ester), salvianolic acid, caffeic acid (hydroxycinnamic acid)	12.8–60.5%	[129]
Lemon balm, *Melissa officinalis* L.	Lamiaceae	EAE	Leaves	Solid/liquid ratio, 1:20; temperature (°), 50; time (hours), 2	Rosmarinic acid (caffeic acid ester), salvianolic acid, caffeic acid (hydroxycinnamic acid).	56.2–65.2%	[129]
Myrtle, *Myrtus communis* L.	Myrtaceae	MAE	Leaves and flowers	Microwave power (W), 400–600; extraction time (s), 30–90; liquid-to-solid ratio (mL/g), 20–40; ethanol proportion (%), 20–100	α-Pinene1,8-cineole, linalool and linalyl acetate (terpene, terpene alcohols)	20– 60%	[101]
Baikal skullcap or Chinese skullcap, *Scutellaria baicalensis* Georgi	Lamiaceae	NADES -MAE	-	The mole ratio of choline chloride to lactic acid, 3:1, 2:1, 1:1, 1:2, 1:3, and 1:4; water content in choline chloride-lactic acid (%), 0, 20, 40, 60, 80.	Baicalin (flavone glycoside) Wogonoside (glycosides of wogonin); Baicalein (flavone) Wogonin (O-methylated flavone)	79.5–84.1%	[46]
Bitter melon, *Momordica charantia* L.	Cucurbitaceae	UAEE	Fruit	Extraction time (min), 30–50; pH, 3–5; enzyme concentration (%), 1.5-2.5	Polysaccharides	29.75% (predicted value 29.8%)	[124]
Asian knotweed, *Polygonum cuspidatum* Siebold & Zucc.	Polygonaceae	UAEE	-	Power (W), 150; temperature (°C), 70; rotation (rpm), 150; frequency (kHz), 40	Resveratrol (stilbenoid)	3.76 mg/g– 11.88 mg/g	[125]
Lemon balm, *Melissa officinalis* L.	Lamiaceae	ASE	Leaves	Temperature (°C), 100; pressure (psi), 1500; static time (min), 5; static cycles, 1; flush volume (%), 60; purge time (s), 120.	Gallic acid, chlorogenic acid, ferulic acid (hydroxycinnamic acids), rosmarinic acid (caffeic acid ester), quercetin (flavonol), rutin (glycoside), apigenin (flavone)	–	[111]
Rosemary, *Rosmarinus officinalis* L., Juniper, *Juniperus communis* ssp. *nana*, Immortelle, *Helichrysum italicum* (Roth) G. Don fil. Mastic tree, *Pistacia lentiscus* L.	Lamiaceae Cupressaceae Asteraceae Anacardiaceae	SFE	Leaves and flower	Pressure (bar), 300; temperature (K), 313; flow rate of CO_2_ (kg/h), 0.4	Verbenone, germacrene D, bornyl acetate, ferruginol, trans-caryophyllene, elemol, γ-cadinene, geraniol or β-eudesmol (terpenes)	2–8%/23.1–50%/10–23%	[98]
Passion flower, *Passiflora alata* Curtis, *P. capsularis, P. cincinnata, P. edulis* f. *flavicarpa, P. edulis* f. *edulis, P. galbana, P. gibertii, P. maliformis, P. malacophylla, P. morifolia, P. mucronata, P. quadrangularis, P. racemosa, P. setacea, P. suberosa P. vitifolia, P. tenuifila*	Passifloraceae	ASE	Leaves	Temperature (°C), 40–80; ethanol concentration (% *w*/*w*), 40–100; number of extraction cycles, 1–5	Isoorientin, orientin, vitexin, isovitexin, rutin (flavone, apigenin flavone glucoside, glycoside)	11.07–47.73%	[114]
Lemon verbena, *Lippia citriodora* Kunth	Verbenaceae	NADES-MAE	Leaves	Choline chloride:lactic acid, 1:2; choline chloride:tartaric acid, 2:1; choline chloride:xylitol, 2:1; choline chloride:fructose:water, 2:1:1; choline chloride:sucrose: water, 4:1:2; **MAE conditions**: Power (W), 700; pressure (bar), 18; temperature (°C), 65; time (min), 20	Gardoside, ixoside, verbascoside, verbascoside, luteolin-7-diglucuronide, apigenin-7-O-diglucuronide (iridoids, phenylpropanoids and flavonoids)	7.25–15.63 mg/g 5.43–9.02 mg/g	[106]
Sage, *Salvia officinalis* L.	Lamiaceae	SFME	-	Soaking 71% water for 1 h; extraction time (min), 20–60; humidity of the matrix (%), 60-80	Odoriferous oxygenated monoterpenes and terpene hydrocarbons	0.85–1.29%/ 0.85–1.59%	[105]
Candy leaf, *Stevia rebaudiana* Bertoni	Asteraceae	PHWE	Leaves	Pressure (Pa), 10.34; static extraction time (min), 5, 10; temperature (°C), 100, 130, 160; cycle number: 1, 2, 3	Total phenolic; condensed tannins; chlorophyll A and B total carotenoid content	5.22–9.33 mg/g 1.37–2.29 mg/g 3.42–3.84 mg/g	[113]
Ginkgo, *Ginkgo biloba* Linn. Ginseng, *Panax ginseng*	Ginkgoaceae Araliaceae	NADES, UAE	Leaves Stems	malic acid:choline chloride, 1:1; malic acid:glucose, 1:1; choline chloride:glucose, 5:2; malic acid:proline, 1:1; glucose:fructose-sucrose, 1:1:1; glycerol:proline:sucrose, 9:4:1; time (min), 30; temperature (°C), 40	Ginkgolides (terpenic lactones) Ginsenosides (panaxosides)	–	[120]
Blackberry, *Morus nigra* L. Wall germander, *Teucrium chamaedrys* L. Bigroot geranium, *Geranium macrorrhizum* L. Comfrey, *Symphytum officinale* L.	Moraceae Lamiaceae Geraniaceae Boraginaceae	SWE	Leaves Flowers Leaves Leaves	Sample to distilled water ratio, 1:40; extraction temperature (°C), 60–200; pressure (bar), 10 extraction time (min), 30	Gallic acid, protocatechuic acid, catechin, chlorogenic acid, caffeic acid (phenolic acids), rutin (glycoside), quercetin (flavonol)	–	[116]
Oregano, *Origanum glandulosum* Desf., Algerian thyme, *Thymus fontanesii* Boiss. & Reut.	Lamiaceae	MAE	Leaves and flowers	Solvent composition, 0, 50, 100% ethanol in water; extraction time (min), 1, 5.5, 10; temperature (°C), 30, 90, 150; microwave extraction reactor, 850 W and 2455 MHz	Gallocatechin (flavan-3-ol) rosmarinic acid (caffeic acid ester)	5.32–18.39%	[100]
Immortelle, *Helichrysum arenarium* L. Moench	Asteraceae	ILMHDE	Inflorescences	Microwave power (W), 120–700	Essential oil; astragalin (3-O-glucoside of kaempferol); quercetin (flavonol); luteolin (flavone); kaempferol (flavonol); apigenin (flavone)	5.12mg/g (essential oil), 3.05mg/g (total flavonoids)	[102]

Abbreviations presented in Table 1.

**Table 5:** Other extraction methods applied to different plant materials and the obtained compounds (in chronological order of the cited works).

Plant Material	Family Name	Extraction Method	Plant Organ	Conditions	Obtained Compounds	Extraction Yield	Ref.
Beetroot, *Beta vulgaris* L., 1753	Pedaliaceae	PEF	Tubers	Nine electric pulses at a constant field strength of 1 kV/cm, with a pulse length of 10 µs; time (min), 60	Betanin (aglycone)	60–80%	[139]
Peppermint, *Mentha piperita* L.	Lamiaceae	OAHD	Fresh aerial parts	Frequency (kHz), 20, 50, 100; intensity (V), 220, 380; time (min), 60	Increased yields of essential oil	2.29–2.58%	[132]
Olive, *Olea europaea* L.	Oleaceae	HVED	Kernel	Needle plate geometry electrodes; voltage (kV), 40; time (µs), 10	Protein, phenolic compounds	555.8–607.5 mg GAE/L	[137]
Sesame, *Sesamum indicum* L.	Pedaliaceae	HVED	Seeds	Disc electrode in the bottom (3.5 cm of diameter) and one needle electrode in the top; voltage (kV), 40; time (µs), 10	Enhanced oil content	4.9–22.4%	[138]
Oregano, *Origanum vulgare* subsp. *viride*	Lamiaceae	OAHD	Dried plant	Intensity (V), 100, 150, 200; temperature (°), 50; time (min), 5	Increased yields of essential oil	9.5–10.6%	[133]

*Abbreviations presented in Table 1.*

**Table 6:** Optimization studies using the response surface method for the production of biologically active compounds from plant materials.

Plant	Family Name	Optimized Process	Process Parameters	Design Method	Independent Variables	Ref.
Lion’s mane mushroom, *Hericium erinaceus* (Bull.) Persoon	Hericiaceae	EAE	Polysaccharides’ yield	BBD	pH, temperature time	[145]
Mongolian milkvetch, *Astragalus membranaceus* Schischkin hairy root cultures	Fabaceae	Optimal biomass production	Isoflavonoids	BBD	Culture temperature, sucrose concentration, inoculum size, and harvest time	[59]
Yarrow, *Achillea millefolium* L., 1753	Asteraceae	MAE	Polyphenolic compounds, flavonoid content, antioxidant activity	CCD	Extraction time, ethanol concentration, liquid/solid ratio, and microwave power	[146]
Woad, *Isatis tinctoria* L. hairy root cultures	Brassicaceae	High speed homogenization coupled with MAE	Alkaloids and flavonoids	BBD	Homogenization time, extraction temperature, microwave power, and extraction time	[77]
Mongolian milkvetch, *Astragalus membranaceus* Schischkin hairy root cultures	Fabaceae	High speed homogenization coupled with CAE	Isoflavonoids	BBD	Negative pressure, homogenization time, liquid/solid ratio, and extraction time	[78]
Golden-and-silver honeysuckle, *Lonicera japonica* Thunb.	Caprifoliaceae	Ultra-turrax based UAE	Organic acids	BBD	Ethanol concentration, time, and liquid/solid ratio	[147]
Candy leaf, *Stevia rebaudiana* (Bertoni)	Asteraceae	MAE	Stevioside rebaudioside-A	CCD	Microwave, power extraction temperature time	[148]
Cumin, *Cuminum cyminum* L.	Apiaceae	MHD	Essential oil	CCD	microwave irradiation time, microwave irradiation power, and moisture content	[149]
Hemp, *Cannabis sativa* L.	Cannabaceae	SFE	Tetrahydrocannabinol	CCD	Pressure, temperature, and co-solvent concentration	[150]
Jimsonweed, *Datura stramonium* hairy roots cultures	Solanaceae	Elicitation	Hyoscyamine	Polynomial models of 3 and 4 degrees	Exposure time and salicylic acid concentration	[144]

*Abbreviations presented in Table 1.*

## References

[1] Murthy H.N., Georgiev M.I., Park S.Y., Dandin V.S., Paek K.Y. (2015). The safety assessment of food ingredients derived from plant cell, tissue and organ cultures: A review. Food Chem..

[2] Croteau R., Kutchan T.M., Lewis N.G., Buchanan B., Gruissem W., Jones R. (2000). Natural products (secondary metabolites). Biochemistry & Molecular Biology of Plants.

[3] (2019). WHO Global Report on Traditional and Complementary Medicine.

[4] Puértolas E., Koubaa M., Barba F.J. (2016). An overview of the impact of electrotechnologies for the recovery of oil and high-value compounds from vegetable oil industry: Energy and economic cost implications. Food Res. Int..

[5] Bucar F., Wube A., Schmid M. (2013). Natural product isolation, how to get from biological material to pure compounds. Nat. Prod. Rep..

[6] Coors A., Brosch M., Kahl E., Khalil R., Michels B., Laub A., Franke K., Gerber B., Fendt M. (2019). *Rhodiola rosea* root extract has antipsychotic-like effects in rodent models of sensorimotor gating. J. Ethnopharmacol..

[7] Luo C., Xu X., Wei X., Feng W., Huang H., Liu H., Xu R., Lin J., Han L., Zhang D. (2019). Natural medicines for the treatment of fatigue: Bioactive components, pharmacology, and mechanisms. Pharmacol. Res..

[8] Allen D., Bilz M., Leaman D.J., Miller R.M., Timoshyna A., Window J. (2014). European Red List of Medicinal Plants.

[9] CORDIS (2014). Final Report Summary, CROPS2INDUSTRY (Non-Food Crops-to-Industry Schemes in EU27). https://cordis.europa.eu/project/rcn/100484/reporting/en.

[10] Cragg G.M., Schepartz S.A., Suffness M., Grever M.R. (1993). The taxol supply crisis. New NCI policies for handling the large-scale production of novel natural product anticancer and anti-HIV agents. J. Nat. Prod..

[11] Knoess W., Wiesner J. (2019). The globalization of traditional medicines: Perspectives related to the European Union regulatory environment. Engineering.

[12] Carvalho A.C.B., Lana T.N., Perfeito J.P.S., Silveira D. (2018). The Brazilian market of herbal medicinal products and the impacts of the new legislation on traditional medicines. J. Ethnopharmacol..

[13] Georgiev M.I., Weber J., Maciuk A. (2009). Bioprocessing of plant cell cultures for mass production of targeted compounds. Appl. Microbiol. Biotechnol..

[14] Lim E.-K., Bowles D. (2012). Plant production systems for bioactive small molecules. Curr. Opin. Biotechnol..

[15] Wilson S.A., Roberts S.C. (2012). Recent advances towards development and commercialization of plant cell culture processes for the synthesis of biomolecules. Plant Biotechnol. J..

[16] Georgiev M.I., Eibl R., Zhong J.J. (2013). Hosting the plant cells in vitro: Recent trends in bioreactors. Appl. Microbiol. Biotechnol..

[17] Georgiev M., Weber J. (2014). Bioreactors for plant cells: Hardware configuration and internal environment optimization as tools for wider commercialization. Biotechnol. Lett..

[18] Eibl R., Meier P., Stutz I., Schildberger D., Huhn T., Eibl D. (2018). Plant cell culture technology in the cosmetics and food industries: Current state and future trends. Appl. Microbiol. Biotechnol..

[19] www.phytonbiotech.com.

[20] Zhong J.J. (2002). Plant cell culture for production of paclitaxel and other taxanes. J. Biosci. Bioeng..

[21] Murthy H.N., Georgiev M.I., Kim Y.-S., Jeong C.-S., Kim S.-J., Paek K.-Y. (2014). Ginsenosides: Prospective for sustainable biotechnological production. Appl. Microbiol. Biotechnol..

[22] Kim H.K., Choi Y.H., Verpoorte R. (2011). NMR based metabolomics: Where do we stand, where do we go?. Trends Biotechnol..

[23] Walsh G. (2010). Biopharmaceutical benchmarks. Nature Biotechnol..

[24] Dos Santos N.V., de Carvalho Santos-Ebinuma V., Pessoa Jr A., Brandão Pereira J.F. (2018). Liquid-liquid extraction of biopharmaceuticals from fermented broth: Trends and future prospects. J. Chem. Technol. Biotechnol..

[25] Stanbury P.F., Whitaker A., Hall S.J., Stanbury P.F., Whitaker A., Hall S.J. (2017). The recovery and purification of fermentation products. Principles of Fermentation Technologym.

[26] Yang Z. (2018). Natural Deep Eutectic Solvents and Their Applications in Biotechnology.

[27] Carta G., Jungbauer A. (2010). Protein Chromatography: Process Development and Scale-Up.

[28] Aguilar O., Rito-Palomares M., Benavides J. (2017). Aqueous two phase systems for the recovery and purification of bioproducts from plants and vegetable tissues. Aqueous Two Phase Systems for Bioprocess Development for the Recovery of Biological Products.

[29] Rosa P.A.J., Azevedo A.M., Sommerfeld S., Bäcker W., Aires-Barros M.R. (2011). Aqueous two phase extraction as a platform in the biomanufacturing industry: Economical and environmental sustainability. Biotechnol. Adv..

[30] Doran P.M. (2005). Bioprocess Engineering Principles.

[31] Riedl W., Raiser T. (2008). Membrane-supported extraction of biomolecules with aqueous two phase systems. Desalination.

[32] Riedl W., Mollet D., Grundle G. (2011). Using Membrane-Supported Liquid–Liquid Extraction for the Measurement of Extraction Kinetics. Chimia.

[33] Bi P.Y., Li D.Q., Dong H.R. (2009). A novel technique for the separation and concentration of penicillin G from fermentation broth: Aqueous two phase flotation. Sep. Purif. Technol..

[34] Tikhomiroff C., Allais S., Klvana M., Hisiger S., Jolicoeur M. (2002). Continuous selective extraction of secondary metabolites from Catharanthus roseus hairy roots with silicon oil in a two-liquid phase bioreactor. Biotechnol. Prog..

[35] Matsumoto M., Ohtani T., Kondo K. (2007). Comparison of solvent extraction and supported liquid membrane permeation using an ionic liquid for concentrating penicillin G. J. Membr. Sci..

[36] Maugeri Z., Domínguez de María P. (2014). Benzaldehyde lyase (BAL)-catalyzed enantioselective C-C bond formation in deep-eutectic solvents− buffer mixtures. J. Mol. Catal. B: Enzym..

[37] Yang Z., Wen Q., Paul B.K., Moulik S.P. (2015). Deep eutectic solvents as a new reaction medium for biotransformations. Ionic Liquid based Surfactant Science: Formulation, Characterization and Applications.

[38] Khodaverdiana S., Dabirmanesh B., Heydarib A., Dashtban-Moghadama E., Khajeha K., Ghazi F. (2018). Activity, stability and structure of laccase in betaine based natural deep eutectic solvents. Int. J. Biol. Macromol..

[39] Francisco M., van den Bruinhorst A., Kroon M.C. (2013). Low-transition temperature mixtures (LTTMs): A new generation of designer solvents. Angew. Chem..

[40] Dai Y., van Spronsen J., Witkamp G.J., Verpoorte R., Choi Y.H. (2013). Natural deep eutectic solvents as new potential media for green technology. Anal. Chim. Acta.

[41] Espino M., de los Ángeles Fernández M., Gomez F.J.V., Boiteux J., Silva M.F. (2018). Green analytical chemistry metrics: Towards a sustainable phenolics extraction from medicinal plants. Microchem. J..

[42] Liu Y., Li J., Fu R., Zhang L., Wang D., Wang D. (2019). Enhanced extraction of natural pigments from Curcuma longa L. using natural deep eutectic solvents. Ind. Crop. Prod..

[43] Chemat F., Vian M.A., Ravi H.K., Khadhraoui B., Hilali S., Perino S., Tixier A.S.F. (2019). Review of Alternative Solvents for Green Extraction of Food and Natural Products: Panorama, Principles, Applications and Prospects. Molecules.

[44] Abbott A.P., Capper G., Davies D.L., Rasheed R.K., Tambyrajah V. (2003). Novel solvent properties of choline chloride/urea mixtures. Chem. Commun..

[45] Zainal-Abidin M.H., Hayyan M., Hayyan A., Jayakumar N.S. (2017). New horizons in the extraction of bioactive compounds using deep eutectic solvents: A review. Anal. Chim. Acta.

[46] Wei Z.F., Wang X.Q., Peng X., Wang W., Zhao C.J., Zu Y.G., Fu Y.J. (2015). Fast and green extraction and separation of main bioactive flavonoids from Radix Scutellariae. Ind. Crops Prod..

[47] Jeong K.M., Ko J., Zhao J., Jin Y., Yoo D.E., Han S.Y., Lee J. (2017). Multi-functioning deep eutectic solvents as extraction and storage media for bioactive natural products that are readily applicable to cosmetic products. J. Clean. Prod..

[48] Espino M., Solari M., de los Ángeles Fernández M., Boiteux J., Gómez M.R., Silva M.F. (2019). Nades-mediated folk plant extracts as novel antifungal agents against *Candida albicans*. J. Pharm. Biomed. Anal..

[49] Espino M., de los Ángeles Fernández M., Gomez F.J.V., Silva M.F. (2016). Natural designer solvents for greening analytical chemistry. Trends Analyt. Chem..

[50] Gutiérrez M.C., Ferrer M.L., Mateo C.R., Monte F.D. (2009). Freeze-drying of aqueous solutions of deep eutectic solvents: A suitable approach to deep eutectic suspensions of self-assembled structures. Langmuir.

[51] Panić M., Elenkov M.M., Roje M., Cvjetko Bubalo M., Radojčić Redovniković I. (2018). Plant-mediated stereoselective biotransformations in natural deep eutectic solvents. Process Biochem..

[52] Yang T.X., Zhao L.Q., Wang J., Song G.L., Liu H.M., Cheng H., Yang Z. (2017). Improving whole cell biocatalysis by addition of deep eutectic solvents and natural deep eutectic solvents. ACS Sustain. Chem. Eng..

[53] Ma X.C., Sun C., Huang S.S., Wang J.K., Zhang B.J., Li F.Y., Wang G., Deng S., Cui J. (2010). Preparative isolation and purification of four prenylflavanones from microbial biotransformation of kurarinone by high-speed counter-current chromatography. Sep. Purif. Technol..

[54] Xia G., Lin C., Liu S. (2016). Tannase-mediated biotransformation assisted separation and purification of theaflavin and epigallocatechin by high speed counter current. Microsc. Res. Tech..

[55] Schwienheer C., Prinz A., Zeiner T., Merz J. (2015). Separation of active laccases from Pleurotus sapidus culture supernatant using aqueous two phase systems in centrifugal partition chromatography. J. Chromatogr. B.

[56] Rincón E., Balu A.M., Luque R., Serrano L. (2019). Mechanochemical extraction of antioxidant phenolic compounds from Mediterranean and medicinal *Laurus nobilis*: A comparative study with other traditional and green novel techniques. Ind. Crop Prod..

[57] Dhanani T., Shah S., Gajbhiye N., Kumar S. (2017). Effect of extraction methods on yield, phytochemical constituents and antioxidant activity of *Withania somnifera*. Arab. J. Chem..

[58] Srivastava S., Cahill D.M., Adholeya A. (2019). Optimal method selection for biocompatible extraction of rosmarinic acid from mycorrhizal hairy roots of *Ocimum basilicum*. Biotechnol. Rep..

[59] Jiao J., Gai Q.Y., Fu J.Y., Ma W., Peng X., Tan S.N., Efferth T. (2014). Efficient production of isoflavonoids by Astragalus membranaceus hairy root cultures and evaluation of antioxidant activities of extracts. J. Agric. Food Chem..

[60] Rodrigues M.F.F., Sousa I.M.O., Vardanega R., Nogueira G.C., Meireles M.A.A., Foglio M.A., Marchese J.A. (2019). Techno-economic evaluation of artemisinin extraction from *Artemisia annua* L. using supercritical carbon dioxide. Ind. Crop. Prod..

[61] Smith R.M. (1999). Supercritical fluids in separation science, The dreams, the reality and the future. J. Chromatogr. A.

[62] Szarka S., Gyurjan I., Laszlo M., Hethelyi E., Kuzovkina I.N., Lemberkovics E., Szoke E. (2010). GC-MS Studies of thiophenes in the supercritical fluid CO_2_ and solvent extracts of *Tagetes patula* L.. Chromatographia.

[63] Anuar N., Markom M., Khairedin S., Johari N.A. (2012). Production and Extraction of Quercetin and (+)-Catechin from *Phyllanthus niruri* Callus Culture. Int. J. Biotechnol. Bioeng..

[64] Thakre N., Prajapati A.K., Mahapatra S.P., Kumar A., Khapre A., Pal D. (2016). Modeling and optimization of reactive extraction of citric acid. J. Chem. Eng. Data.

[65] Djas M., Henczka M. (2016). Reactive extraction of citric acid using supercritical carbon dioxide. J. Supercrit. Fluids.

[66] Afzal W., Liu X., Prausnitz J.M. (2014). High solubilities of carbon dioxide in tetraalkyl phosphonium based ionic liquids and the effect of diluents on viscosity and solubility. J. Chem. Eng. Data.

[67] Henczka M., Djas M. (2018). Reactive extraction of succinic acid using supercritical carbon dioxide. Sep. Sci. Technol..

[68] Kursinszki L., Hank H., László I., Szőke É. (2005). Simultaneous analysis of hyoscyamine, scopolamine, 6β-hydroxyhyoscyamine and apoatropine in Solanaceous hairy roots by reversed phase high-performance liquid chromatography. J Chromatogr. A.

[69] Wahby I., Arráez-Román D., Segura-Carretero A., Ligero F., Caba J.M., Fernández-Gutiérrez A. (2006). Analysis of choline and atropine in hairy root cultures of *Cannabis sativa* L. by capillary electrophoresis-electrospray mass spectrometry. Electrophoresis.

[70] Hahn T., Lang S., Ulber R., Muffler K. (2012). Novel procedures for the extraction of fucoidan from brown algae. Process Biochem..

[71] Kadam S.U., Tiwari B.K., O’Donnell C.P. (2013). Application of novel extraction technologies for bioactives from marine algae. J. Agric. Food Chem..

[72] Cassol L., Rodrigues E., Pelayo C., Noreña Z. (2019). Extracting phenolic compounds from *Hibiscus sabdariffa* L. calyx using microwave assisted extraction. Ind. Crop. Prod..

[73] Chemat F., Rombaut N., Meullemiestre A., Turk M., Perino S., Fabiano-Tixier A.S., Abert-Vian M. (2017). Review of green food processing techniques. Preservation, transformation, and extraction. Innov. Food Sci. Emerg. Technol..

[74] Gao M., Song B.Z., Liu C.Z. (2006). Dynamic microwave assisted extraction of flavonoids from Saussurea medusa Maxim cultured cells. Biochem. Eng. J..

[75] Lee J.Y., Kim J.H. (2011). Development and optimization of a novel simultaneous microwave assisted extraction and adsorbent treatment process for separation and recovery of paclitaxel from plant cell cultures. Sep. Purif. Technol..

[76] Romanik G., Gilgenast E., Przyjazny A., Kamiński M. (2007). Techniques of preparing plant material for chromatographic separation and analysis. J. Biochem. Biophys. Methods.

[77] Jiao J., Gai Q.Y., Zhang L., Wang W., Luo M., Zu Y.G., Fu Y.J. (2015). High-speed homogenization coupled with microwave assisted extraction followed by liquid chromatography-tandem mass spectrometry for the direct determination of alkaloids and flavonoids in fresh *Isatis tinctoria* L. hairy root cultures. Anal. Bioanal. Chem..

[78] Jiao J., Gai Q.Y., Luo M., Peng X., Zhao C.J., Fu Y.J., Ma W. (2015). Direct determination of astragalosides and isoflavonoids from fresh Astragalus membranaceus hairy root cultures by high speed homogenization coupled with cavitation-accelerated extraction followed by liquid chromatography-tandem mass spectrometry. RSC Adv..

[79] Li S.M., Fu Y.J., Zu Y.G., Zu B.S., Wang Y., Efferth T. (2009). Determination of paclitaxel and its analogues in the needles of Taxus species by using negative pressure cavitation extraction followed by HPLC-MS-MS. J. Sep. Sci..

[80] Liu W., Fu Y.J., Zu Y.G., Kong Y., Zhang L., Zu B.S., Efferth T. (2009). Negative-pressure cavitation extraction for the determination of flavonoids in pigeon pea leaves by liquid chromatography-tandem mass spectrometry. J. Chromatogr. A.

[81] Krause J., Merz J. (2017). Comparison of enzymatic hydrolysis in a centrifugal partition chromatograph and stirred tank reactor. J. Chromatogr. A.

[82] Song X., Cui L., Li J., Yan H., Li L., Wen L., Geng Y., Wang D. (2019). A novel bioreactor for highly efficient biotransformation of resveratrol from polydatin with high-speed counter-current chromatography. LWT Food Sci. Technol..

[83] Wang D., Zhao H., Zhu H., Wen L., Yu J., Li L., Chen L., Geng Y. (2019). A novel method for highly efficient biotransformation and separation of isoflavone aglycones from soybean with high-speed counter-current chromatography. Ind. Crops Prod..

[84] Wang D., Khan M.S., Cui L., Song X., Zhu H., Ma T., Li X., Sun R. (2019). A novel method for the highly efficient biotransformation of genistein from genistin using a high-speed counter-current chromatography bioreactor. RSC Adv..

[85] Krause J., Oeldorf T., Schembecker G., Merz J. (2015). Enzymatic hydrolysis in an aqueous organic two phase system using centrifugal partition chromatography. J. Chromatogr. A.

[86] Nioi C., Riboul D., Destrac P., Marty A., Marchal L., Condoret J.S. (2015). The centrifugal partition reactor, a novel intensified continuous reactor for liquid=liquid enzymatic reactions. Biochem. Eng. J..

[87] Berthod A., Carda-Broch S. (2004). Use of ionic liquid 1-butyl-3-methylimidazolium hexafluorophosphate in countercurrent chromatography. Anal. Bioanal. Chem..

[88] Ruiz-Angel M.J., Pino V., Carda-Broch S., Berthod A. (2007). Solvent systems for countercurrent chromatography: An aqueous two phase liquid system based on a room temperature ionic liquid. J. Chromatogr. A.

[89] Handa S.S., Handa S.S., Khanuja S.P.S., Longo G., Rakesh D.D. (2008). An overview of extraction techniques for medicinal and aromatic plants. Extraction Technologies for Medicinal and Aromatic Plants.

[90] Ghafoor K., Al-Juhaimi F.Y., Choi Y.H. (2012). Supercritical fluid extraction of phenolic compounds and antioxidants from grape (*Vitis labrusca* B.) seeds. Plant Foods Hum. Nutr..

[91] Wang L., Wu M., Liu H.M., Ma Y.X., Wang X.D., Qin G.Y. (2017). Subcritical Fluid Extraction of Chinese Quince Seed: Optimization and Product Characterization. Molecules.

[92] Toubane A., Rezzoug S.A., Besombes C., Daoud K. (2017). Optimization of accelerated solvent extraction of Carthamus caeruleus L. Evaluation of antioxidant and anti-inflammatory activity of extracts. Ind. Crops Prod..

[93] Fang X., Wang J., Hao J., Li X., Guo N. (2015). Simultaneous extraction, identification and quantification of phenolic compounds in *Eclipta prostrata* using microwave assisted extraction combined with HPLC-DAD-ESI-MS/MS. Food Chem..

[94] Rodríguez-Solana R., Vázquez-Araújo L., Salgado J.M., Domínguez J.M., Cortés-Diéguez S. (2016). Optimization of the process of aromatic and medicinal plant maceration in grape marc distillates to obtain herbal liqueurs and spirits. J. Sci. Food Agric..

[95] Luque de Castro M.D., Priego-Capote F. (2010). Soxhlet extraction: Past and present panacea. J. Chromatogr. A.

[96] Roby M.H.H., Sarhan M.A., Selim K.A.H., Khalel K.I. (2013). Evaluation of antioxidant activity, total phenols and phenolic compounds in thyme (*Thymus vulgaris* L.), sage (*Salvia officinalis* L.), and marjoram (*Origanum majorana* L.) extracts. Ind. Crops Prod..

[97] Ahmad R., Ahmad N., Shehzad A. (2019). Solvent and temperature effects of accelerated solvent extraction (ASE) with Ultra-high-pressure liquid chromatography (UHPLC-PDA) technique for determination of Piperine and its ICP-MS analysis. Ind. Crop. Prod..

[98] Mouahid A., Dufour C., Badens E. (2017). Supercritical CO_2_ extraction from endemic Corsican plants; comparison of oil composition and extraction yield with hydrodistillation method. J. CO_2_ Util..

[99] Du F.Y., Xiao X.H., Luo X.J., Li G.K. (2009). Application of ionic liquids in the microwave assisted extraction of polyphenolic compounds from medicinal plants. Talanta.

[100] Nabet N., Gilbert-López B., Madani K., Herrero M., Ibáñez E., Mendiola J.A. (2019). Optimization of microwave assisted extraction recovery of bioactive compounds from *Origanum glandulosum* and *Thymus fontanesii*. Ind. Crops Prod..

[101] Dahmoune F., Nayak B., Moussi K., Remini H., Madani K. (2015). Optimization of microwave- assisted extraction of polyphenols from *Myrtus communis* L. leaves. Food Chem..

[102] Liu X., Jing X., Li G. (2019). A process to acquire essential oil by distillation concatenated liquid-liquid extraction and flavonoids by solid-liquid extraction simultaneously from *Helichrysum arenarium* (L.) Moench inflorescences under ionic liquid microwave mediated. Sep. Purif. Technol..

[103] Liu T., Sui X., Lei R.Z., Yuangang Y., Zhang Z.L., Zhang Y., Zhang Z. (2011). Application of ionic liquids based microwave assisted simultaneous extraction of carnosic acid, rosmarinic acid and essential oil from *Rosmarinus officinalis*. J. Chromatogr. A.

[104] Liu Y., Yang L., Zu Y., Zhao C., Zhang L., Zhang Y., Zhang Z., Wang W. (2012). Development of an ionic liquid based microwave assisted method for simultaneous extraction and distillation for determination of proanthocyanidins and essential oil in *Cortex cinnamomic*. Food Chem..

[105] Wei Z.F., Zhao R.N., Dong L.J., Zhao X.Y., Su J.X., Zhao M., Li L., Bian Y.J., Zhang L.J. (2018). Dual-cooled solvent-free microwave extraction of *Salvia officinalis* L. essential oil and evaluation of its antimicrobial activity. Ind. Crops Prod..

[106] Ivanović M., Alañón M.E., Arráez-Román D., Segura-Carretero A. (2018). Enhanced and green extraction of bioactive compounds from *Lippia citriodora* by tailor-made natural deep eutectic solvents. Food Res. Int..

[107] Chizoba Ekezie F.G., Sun D.W., Cheng J.H. (2017). Acceleration of microwave assisted extraction processes of food components by integrating technologies and applying emerging solvents: A review of latest developments. Trends Food Sci. Technol..

[108] Yu Y., Chen B., Chen Y., Xie M., Duan H., Li Y., Duan G. (2009). Nitrogen-protected microwave assisted extraction of ascorbic acid from fruit and vegetables. J. Sep. Sci..

[109] Nieto A., Borrull F., Pocurull E., Marcé R.M. (2010). Pressurized liquid extraction: A useful technique to extract pharmaceuticals and personal-care products from sewage sludge. Trends Anal. Chem..

[110] Panja P. (2018). Green extraction methods of food polyphenols from vegetable materials. Curr. Opin. Food Sci..

[111] Fierascu I., Georgiev M.I., Ortan A., Fierascu R.C., Avramescu S.M., Ionescu D., Sutan A., Brinzan A., Ditu L.M. (2017). Phyto-mediated metallic nano-architectures via *Melissa officinalis* L.: Synthesis, characterization and biological properties. Sci. Rep..

[112] Plaza M., Turner C. (2015). Pressurized hot water extraction of bioactives. Trends Anal. Chem..

[113] Bursać Kovačević D., Barba F.J., Granato D., Galanakis C.M., Herceg Z., Dragović- Uzelac V., Putnik P. (2018). Pressurized Hot Water Extraction (PHWE) for the green recovery of bioactive compounds and steviol glycosides from *Stevia rebaudiana* Bertoni Leaves. Food Chem..

[114] Gomes S.V.F., Portugal L.A., dos Anjos J.P., de Jesus O.N., de Oliveira E.J., David J.P., David J.M. (2017). Accelerated solvent extraction of phenolic compounds exploiting a Box-Behnken design and quantification of five flavonoids by HPLC-DAD in *Passiflora* species. Microchem. J..

[115] Herrero M., Plaza M., Cifuentes A., Ibáñez E. (2010). Green processes for the extraction of bioactives from Rosemary: Chemical and functional characterization via ultra-performance liquid chromatography-tandem mass spectrometry and in-vitro assays. J. Chromatogr. A.

[116] Nastić N., Švarc-Gajić J., Delerue-Matos C., Barroso M.F., Soares C., Moreira M.M., Morais S., Mašković P., Srček V.G., Slivac I. (2018). Subcritical water extraction as an environmentally-friendly technique to recover bioactive compounds from traditional Serbian medicinal plants. Ind. Crops Prod..

[117] Carr A.G., Mammucari R., Foster N.R. (2011). A review of subcritical water as a solvent and its utilisation for the processing of hydrophobic organic compounds. Chem. Eng. J..

[118] Arapitsas P., Turner C. (2008). Pressurized solvent extraction and monolithic column-HPLC/DAD analysis of anthocyanins in red cabbage. Talanta.

[119] Lupacchini M., Mascitti A., Giachi G., Tonucci L., d’Alessandro N., Martinez J., Colacino E. (2017). Sonochemistry in non-conventional, green solvents or solvent free reactions. Tetrahedron.

[120] Liu X., Ahlgren S., Korthout H.A.A.J., Salomé-Abarca L.F., Bayona L.M., Verpoorte R., Choi Y.H. (2018). Broad range chemical profiling of natural deep eutectic solvent extracts using a high performance thin layer chromatography-based method. J. Chromatogr. A.

[121] Roselló-Soto E., Parniakov O., Deng Q., Patras A., Koubaa M., Grimi N., Boussetta N., Tiwari B.K., Vorobiev E., Lebovka N. (2016). Application of non-conventional extraction methods: Toward a sustainable and green production of valuable compounds from mushrooms. Food Eng. Rev..

[122] Jia X.J., Zhang C., Qiu J.F., Wang L.L., Bao J.L., Wang K., Zhang Y.L., Chen M.W., Wan J.B., Su H.X. (2015). Purification, structural characterization and anticancer activity of the novel polysaccharides from *Rhynchosia minima* root. Carbohydr. Polym..

[123] Fernando P.S., Sanjeewa K.K.A., Samarakoon K.W., Lee W.W., Kim H.S., Kang N., Ranasinghe P., Lee H.S., Jeon Y.J. (2017). A fucoidan fraction purified from *Chnoospora minima*; a potential inhibitor of LPS-induced inflammatory responses. Int. J. Biol. Macromol..

[124] Fan T., Hu J.G., Fu L.D., Zhang L.J. (2015). Optimization of enzymolysis-ultrasonic assisted extraction of polysaccharides from *Momordica charantia* L. by response surface methodology. Carbohydr. Polym..

[125] Lin J.A., Kuo C.H., Chen B.Y., Li Y., Liu Y.C., Chen J.H., Shieh C.J. (2016). A novel enzyme assisted ultrasonic approach for highly efficient extraction of resveratrol from *Polygonum cuspidatum*. Ultrason. Sonochem..

[126] Azmir J., Zaidul I.S.M., Rahman M.M., Sharif K.M., Mohamed A., Sahena F., Jahurul M.H.A., Ghafoor K., Norulaini N.A.N., Omar A.K.M. (2013). Techniques for extraction of bioactive compounds from plant materials: A review. J. Food Eng..

[127] Chen S., Xing X.H., Huang J.J., Xu M.S. (2011). Enzyme assisted extraction of flavonoids from *Ginkgo biloba* leaves: Improvement effect of flavonol transglycosylation catalyzed by *Penicillium decumbens* cellulase. Enzyme Microb. Technol..

[128] Puri M., Sharma D., Barrow C.J. (2012). Enzyme assisted extraction of bioactives from plants. Trends Biotechnol..

[129] Miron T.L., Herrero M., Ibáñez E. (2013). Enrichment of antioxidant compounds from lemon balm (*Melissa officinalis*) by pressurized liquid extraction and enzyme assisted extraction. J. Chromatogr. A.

[130] Knirsch M.C., dos Santos C.A., Martins de Oliveira A.A., Vicente S., Vessoni Penna T.C. (2010). Ohmic heating, a review. Trends Food Sci. Technol..

[131] Gavahian M., Farahnaky A. (2018). Ohmic assisted hydrodistillation technology: A review. Trends Food Sci. Technol..

[132] Gavahian M., Farhoosh R., Javidnia K., Shahidi F., Farahnaky A. (2015). Effect of applied voltage and frequency on extraction parameters and extracted essential oils from *Mentha piperita* by ohmic assisted hydrodistillation. Innov. Food Sci. Emerg. Technol..

[133] Hashemi S.M.B., Nikmaram N., Esteghlal S., Khaneghah A.M., Niakousari M., Barba F.J., Roohinejad S., Koubaa M. (2017). Efficiency of Ohmic assisted hydrodistillation for the extraction of essential oil from oregano (*Origanum vulgare* subsp. *viride*) spices. Innov. Food Sci. Emerg. Technol..

[134] Locke B.R., Sato M., Sunka P., Hoffmann M.R., Chang J.S. (2006). Electrohydraulic discharge and nonthermal plasma for water treatment. Ind. Eng. Chem. Res..

[135] Martynenko A., Kudra T. (2016). Electrically-induced transport phenomena in EHD drying, A review. Trends Food Sci. Technol..

[136] Dalvi-Isfahan M., Hamdami N., Le-Bail A., Xanthakis E. (2016). The principles of high voltage electric field and its application in food processing: A review. Food Res. Int..

[137] Roselló-Soto E., Barba F.J., Parniakov O., Galanakis C.M., Lebovka N., Grimi N., Vorobiev E. (2015). High voltage electrical discharges, pulsed electric field, and ultrasound assisted extraction of protein and phenolic compounds from olive kernel. Food Bioproc. Tech..

[138] Sarkis J.R., Boussetta N., Tessaro I.C., Marczak L.D.F., Vorobiev E. (2015). Application of pulsed electric fields and high voltage electrical discharges for oil extraction from sesame seeds. J. Food Eng..

[139] Fincan M., De Vito F., Dejmek P. (2004). Pulsed electric field treatment for solid-liquid extraction of red beetroot pigment. J. Food Eng..

[140] Guderjan M., Töpfl S., Angersbach A., Knorr D. (2005). Impact of pulsed electric field treatment on the recovery and quality of plant oils. J. Food Eng..

[141] Pina-Pérez M.C., Rivas A., Martínez A., Rodrigo D. (2018). Effect of thermal treatment, microwave, and pulsed electric field processing on the antimicrobial potential of açaí (*Euterpe oleracea*), stevia (*Stevia rebaudiana* Bertoni), and ginseng (*Panax quinque* folius L.) extracts. Food Control.

[142] Dahmoune F., Remini H., Dairi S., Aoun O., Moussi K., Bouaoudia-Madi N., Adjeroud N., Kadri N., Lefsih K., Boughani L. (2015). Ultrasound assisted extraction of phenolic compounds from P. lentiscus L. leaves: Comparative study of artificial neural network (ANN) versus degree of experiment for prediction ability of phenolic compounds recovery. Ind. Crop. Prod..

[143] Wong K.H., Li G.Q., Li K.M., Razmovski-Naumovski V., Chan K. (2017). Optimisation of Pueraria isoflavonoids by response surface methodology using ultrasonic assisted extraction. Food Chem..

[144] Amdoun R., Benyoussef E.H., Benamghar A., Khelifi L. (2019). Prediction of hyoscyamine content in *Datura stramonium* L. hairy roots using different modeling approaches: Response Surface Methodology (RSM), Artificial Neural Network (ANN) and Kriging. Biochem. Eng. J..

[145] Zhu Y., Li Q., Mao G., Zou Y., Feng W., Zheng D., Wang W., Zhou L., Zhang T., Yang J. (2014). Optimization of enzyme assisted extraction and characterization of polysaccharides from *Hericium erinaceus*. Carbohydr. Polym..

[146] Milutinović M., Radovanović N., Ćorović M., Šiler-Marinković S., Rajilić-Stojanović M., Dimitrijević-Branković S. (2015). Optimisation of microwave assisted extraction parameters for antioxidants from waste Achillea millefolium dust. Ind. Crops Prod..

[147] Xu W.J., Zhai J.W., Cui Q., Liu J.Z., Luo M., Fu Y.J., Zu Y.G. (2016). Ultra-turrax based ultrasound assisted extraction of five organic acids from honeysuckle *Lonicera japonica* Thunb. and optimization of extraction process. Sep. Purif. Technol..

[148] Ameer K., Bae S.W., Jo Y., Lee H.G., Ameer A., Kwon J.H. (2017). Optimization of microwave assisted extraction of total extract, stevioside and rebaudioside-A from *Stevia rebaudiana* (Bertoni) leaves, using response surface methodology (RSM) and artificial neural network (ANN) modelling. Food Chem..

[149] Benmoussa H., Elfalleh W., He S., Romdhane M., Benhamou A., Chawech R. (2018). Microwave hydrodiffusion and gravity for rapid extraction of essential oil from Tunisian cumin (*Cuminum cyminum* L.) seeds: Optimization by response surface methodology. Ind. Crops Prod..

[150] Gallo-Molina A.C., Castro-Vargasa H.I., Garzón-Méndez W.F., Martínez Ramírez J.A., Rivera Monroy Z.J., King J.W., Parada-Alfonso F. (2019). Extraction, isolation and purification of tetrahydrocannabinol from the *Cannabis sativa* L. plant using supercritical fluid extraction and solid phase extraction. J. Supercrit. Fluids.

[151] Pilkington J.L., Preston C., Gomes R.L. (2014). Comparison of response surface methodology (RSM) and artificial neural networks (ANN) towards efficient extraction of artemisinin from *Artemisia annua*. Ind. Crops Prod..

